# Reassessing sepsis research: new clues for old players and new players for an old symptom to improve patient outcomes

**DOI:** 10.17179/excli2025-8514

**Published:** 2025-08-28

**Authors:** Jean-Marc Cavaillon, Jean Carlet

**Affiliations:** 1 Institut Pasteur, Paris, France; 2 [Intensivist, outgoing President of WAAAR (World Alliance Against Antibiotic Resistance), past-president of the European Society of Intensive Care Medicine" (ESICM), past-member of International Sepsis Forum] Créteil, France

**Keywords:** antibiotics, apoptosis, compartmentalization, cytokines, diagnosis, microbiome

## Abstract

Sepsis remains a global health problem that causes millions of deaths each year. A rapid and accurate diagnosis is highly desired to allow a rapid use of appropriate antibiotics. A better understanding of the associated pathophysiology has been achieved these recent years. The initial appropriate immune response to infection evolves towards an overwhelmed inflammatory response involving both pro- and anti-inflammatory players that act concomitantly. It also includes cell deaths and cellular dysfunctions of leukocytes, endothelial cells and epithelial cells, associated with mitochondrial dysfunction. These dysregulations are responsible for organ impairment and alteration of immune status of circulating leukocytes. In contrast, within the tissues, an over-activation exists as illustrated by transcriptomic analyses of organs of patients deceased of sepsis, and revealed by the presence of a macrophage activation syndrome within the bone marrow. Despite progresses in understanding the mechanisms underlying sepsis and despite successful therapies in animal models, no real new therapies have emerged these recent decades. This failure may reflect the yin yang aspect of the same players of the host response such as fever, release of cytokines, or coagulation which can display both a beneficial or a detrimental role. Great hopes are now expected from precision medicine, based on patients' endotypes which should help to decipher the patient's sub-groups who could benefit from the different treatments, or to define some appropriate time windows for a given treatment.

See also the graphical abstract(Fig. 1).

## Introduction

Between 2000 and 2024, the number of articles on sepsis or septic shock published per year in the scientific press has been multiplied by 4.4. It illustrates the tremendous efforts of the medical and scientific communities to better decipher this syndrome, to further understand its pathophysiology, to better define the respective roles of the cells and the mediators, to better diagnose it, and eventually to better treat the patients. The specific increase of publications observed in 2020 reflects that the severe cases of SARS-Cov-2 infection were classified as viral sepsis. It also illustrates that sepsis remains a challenge of public health as pointed out by the WHO statement on May, 29^th^ 2017, recognizing sepsis as a global health priority (Reinhart et al. 2017[250]). To avoid sepsis, hygiene was advocated as soon as 1795 by Alexander Gordon in Aberdeen, and demonstrated by Ignaz Semmelweis in 1847 in Vienna, while the presence of circulating bacteria was identified in 1869 by two Alsatian physicians, Victor Feltz and Léon Coze, in the blood of a patient who died of puerperal sepsis (Cavaillon & Chrétien, 2019[55]). Soon it appears that the death consecutive to sepsis, while initiated by pathogens and/or its derived toxins, was the consequence of an overwhelmed response of the organism ending in organ failure. This was beautifully summed up in this statement *“Except on few occasions, the patient appears to die from the body's response to infection rather than from it" *assigned to Sir William Osler who would have written such a statement in his book “The Evolution of Modern Medicine” (1913). But a careful analysis of his text does not allow to find such a declaration!

## At the Beginning are the Pathogens

### Viruses, fungi and bacteria 

Before the COVID-19 pandemic, bacteria were considered as the main causes of sepsis. But, severe forms of COVID-19 were fulfilling the definitions of sepsis (Li et al., 2020[180]; Vincent, 2021[321]). Similarly, severe cases of infections by Middle East respiratory syndrome coronavirus; influenza, chikungunya, dengue, hemorrhagic fever, and Ebola viruses display similarities with bacterial sepsis (Xu et al. 2024[346]; Gürtler et al. 2024[125]). The nature of the most frequent bacteria varies depending on the countries, the age of the patients (e.g. neonatal sepsis *versus* senior citizens), the site of infection (urinary, pulmonary, intra-abdominal, skin, catheter…) (Rhee et al. 2020[254]).* Escherichia coli, Staphylococcus aureus, streptococcus pneumoniae, Klebsiella pneumoniae, Enterococcus, Acinetobacter baumannii,* and* Pseudomonas aeruginosa *remain the most frequent invaders. 

Despite the concentrations of inflammatory factors (e.g. C reactive protein (CRP); procalcitonin (PCT); tumor necrosis factor (TNF)) in sepsis caused by Gram-negative bacteria were reported to be higher than in sepsis caused by Gram-positive bacteria, the two groups had no significant difference in terms of survival rate, coagulation function, or hospital stay (Tang et al. 2023[308]). 

Among the different pathogens, the presence of virulence genes has a significant impact on severity and outcome. For example, in *E. coli* bacteremia, the presence of cytotoxic necrotizing factor-1 gene *(cnf*) increased the risk of severe illness by 6.75-fold and β-lactamase gene (*bla_TEM_*) by 2.59-fold. The presence of ferric yersiniabactin uptake receptor gene was associated with an increased mortality (Odds Ratio = 8.05), while the presence of P fimbriae genes had a protective role (Odds Ratio = 0.094) (Mora-Rillo et al. 2015[214]). More recently the list of virulence factors was further defined in *E. coli* isolates from sepsis patients. One virulence gene (*kpsMII_K23*), was significantly more common in isolates of patients who died, and *kpsMII_K23* and *cvaC *(Microcin C) were significantly more frequent in isolates of patients who were admitted to the ICU. Fourteen virulence genes significantly differed between patients with and without sepsis (D'Onofrio et al. 2023[93]). Among the virulence factors, bacterial exotoxins, and endotoxins contribute to the pathogenesis of sepsis as potent inducers of cytokines. An overzealous production, leading up to a cytokine storm, can be deleterious and contributes to mortality consecutive to sepsis (Cavaillon, 2018[48]). Of note, endotoxin can translocate from the gut as it has been reported in patients after abdominal surgery or after cardiac arrest (Cabié et al. 1993[34]; Adrie et al. 2002[3]). All those toxins have probably a key role in the development of sepsis (Sriskandan et al. 1996[290]), but, apart from endotoxin, they have been poorly studied and efforts should be deployed to learn more about their role.

The frequency of fungal sepsis remains low. In a South Korean study, fungal pathogens were identified in 3.4 % of patients with sepsis. Among those patients, 38.3 % had co-existing hematologic or solid organ cancer. Fungal pathogens were associated with chronic kidney disease, and observed in immunocompromised patients. The most common fungal pathogens were *Candida albicans *(47.9 %), *Candida glabrata* (20.6 %), and *Candida tropicalis* (13.5 %). In this study, intensive care unit (ICU) mortality was 30 % (Lee et al. 2025[174]). However, survival has been regularly reported to be lower in patients with bloodstream infections consecutive to fungal organisms (Guo et al. 2023[124]). In a global report concerning 120 countries, an estimate 1 565 000 people have a *Candida* bloodstream infection or invasive candidiasis each year, with a 63.6 % mortality (Denning 2024[89]).

### Antimicrobial resistance (AMR)

"*Unless the many concerned players act urgently in a coordinated way, the world is moving towards a post-antibiotic era in which common infections and minor injuries, which were treated for decades, could kill again*" Dr. Keiji Fukuda, Assistant Director-General of WHO, April 30, 2014.

Prevalence of antibiotic resistance concerns more than 25 % of patients with sepsis (Rhee et al. 2020[254]), while the global burden of bacterial antimicrobial resistance (AMR) is associated with directly attributable death (Antimicrobial Resistance Collaborators, 2022[10]). Of note, death associated and attributable with antimicrobial resistance has decreased between 1990 and 2021 for the neonatal sepsis and the youngest patients (< 9 years old) while it has significantly increased for the adults (> 45 years old) and particularly for the aged population (> 65 years old) (GBD 2021 Antimicrobial Resistance Collaborators, 2024[113]). In 2021, an estimated 4·71 million deaths were associated with bacterial antimicrobial resistance, including 1.14 million deaths attributable to bacterial AMR. A forecast to 2050 ends to an estimation of 8.22 million deaths associated with AMR and 1.91 million deaths attributable to AMR. However, it is very difficult to differentiate patients who die from AMR, of those who die with AMR. Anti-microbial resistance is also very different from country to country. For example, resistance of *Klebsiella pneumoniae* to third generation cephalosporins goes from 0.0 % in Finland, up to 80.4 % in Greece (ECDC, 2023[96]). It might also reflect the restricted access to antibiotics with prescriptions in some countries while a free access in developing countries favors the emergence of AMR. 

A direct relationship between AMR and mortality has been illustrated in different settings, and in different countries. An increased mortality rate associated with antibiotic resistance was observed for all bacterial species with the exception of Gram-positive cocci, and remained significant or near-significant regardless of the French hospital ward (Guillemot et al. 2005[123]). In Addis Abeba (Ethiopia), the mortality of patients with blood stream infection was four times higher in those with resistance to 3^rd^ generation cephalosporins (Seboxa et al. 2015[266]). In Taipei (Taiwan), the overall in-hospital mortality rate was with 23.3 % in patients with bacteremia caused by methicillin sensitive *S. aureus*, but 47.5 % in patients with healthcare associated methicillin resistant *S. aureus* (Wang et al. 2015[327]). The methodology of many studies had serious methodologic weaknesses. Moreover, some other studies showed a very modest attributable mortality to AMR. In conclusion of this serious issue, it seems that AMR might induce some degree of over-mortality, but this effect has probably been over-estimated (Carlet & Chevret, Submitted[40]).

Of note, the emergence of AMR can be affected by other parameters, such as climate change and increased temperature (Li et al, 2022[184]; Magnano San Lio et al. 2023[196]). Nevertheless, one should not forget that AMR is a natural process among bacteria to defend themselves against other prokaryotes. As illustrated by the study of bacteria isolated from a 13,000 year-old cave ice core, among 68 bacterial isolates, more than 50 % of the strains displayed a high resistance to 17 antibiotics species (Paun et al. 2021[237]). Because AMR has to be efficiently tackled, numerous new tracks are offered which include new-generation antibiotics, human monoclonal antibodies, bacteriophages, antimicrobial peptides, and gene therapy (Ho et al. 2025[131]).

### Detection: the hunt for a rapid and trustable diagnostic test

Diagnosis of sepsis offers different challenges. A rapid identification of the causative bacteria and their antimicrobial susceptibilities are highly desired to offer to the patients the most appropriate antibiotic(s), which remain the most effective drugs during sepsis (Vallés et al. 2003[316]). Many efforts have been made to offer the fastest tools which will avoid dependence on a too long time-consuming blood cultures (Choi et al. 2010[69]; Ganguli et al. 2022[108]). New smart technologies are proposed to capture bacteria within the bloodstream (Kim et al. 2024[165]; Abafogi et al. 2024[1]) allowing a further rapid PCR analysis for identification. The identification of four sepsis-causing pathogens (*E.coli, K. pneumoniae, S. aureus and S. pyogenes*) has been achieved with a genomic, transcriptomic, proteomic and metabolomic technological framework (Mu et al. 2023[216]).

However, a systematic review and meta-analysis conducted on rapid molecular assays concluded that their low sensitivity means that they cannot replace blood culture. However, this analysis considered that these assays may have value as an add-on test by increasing pathogen detection rates. The authors suggested that higher-sensitivity assays were needed which could possibly be achieved by expanding pathogen coverage and increasing blood sample volumes (Rapszky et al. 2025[247]). But a difficulty is linked to the desired high sensitivity of the test. Indeed, the presence of bacteria in blood of 7.9 % healthy controls and that of viruses in blood of 72.3 % healthy controls (Shah et al. 2023[272]) is a challenge rarely addressed to ensure that a too high sensitivity may not be associated with false positivity. As a matter of fact, the notion of blood as a sterile environment has been challenged by findings demonstrating the presence of a microbiome in healthy people (Moriyama et al. 2008[215]; Castillo et al. 2019[46]; D'Aquila et al; 2012[79]), and its possible association with disease states (Chen et al. 2024[66]; Khan et al. 2025[163]). Other authors do not sustain the hypothesis of a consistent core microbiome endogenous to human blood, but rather support a transient and sporadic translocation of commensal microbes from other body sites into the bloodstream (Tan et al. 2023[306]).

Other tools are developed to identify patients with sepsis among at risk patients using host response biomarkers. In 2020, 258 biomarkers of sepsis had been listed (Pierrakos et al. 2020[243]). Taken individually, acute phase proteins, tissue injury biomarkers, damage associated molecular patterns (DAMPs), cytokines, chemokines, hormones, apoptosis-related biomarkers, soluble receptors, enzymes, coagulation biomarkers, vascular endothelial biomarkers, metabolites, and leukocyte-surface biomarkers, although of putative interest, remain of limited predictive values (Parlato & Cavaillon 2015[233]). Despite C-reactive protein (CRP), procalcitonin (PCT), or serum amyloid A (SAA) are regularly claimed to allow diagnosis of sepsis, their areas under the curve (AUC) of their ROC curve remain modest (≤ 0.82) (Li et al. 2024[183]). Despite being widely spread and still popular, a 2007 systematic review and meta-analysis revealed that procalcitonin cannot reliably differentiate sepsis from other non-infectious causes of systemic inflammatory response syndrome in critically ill adult patients (Tang et al. 2007[309]). In a study aimed to distinguish between patients, local bacterial infection and bloodstream infections, the AUC of CRP, PCT, IL-6 and IL-10 were respectively, 0.59, 0.83, 0.73 and 0.86, thus of rather limited use (Yang et al. 2023[350]). According to our own experiences, the challenge to decipher the occurrence of sepsis among ICU patients might be far greater than among patients in emergency departments (Parlato et al. 2018[234]; Velly et al. 2021[319]). Indeed, deciphering the occurrence of an infection in ICU patients with a high inflammatory background consecutive to any types of different sterile insults, renders the approach more difficult even when using a combination of plasma and cell surface markers. An interesting approach was to sample the patients in the ambulance to measure metabolomic and lipidomic markers with the aim to early identify sepsis patients (Salihovic et al. 2024[260]). A similar early sampling for trauma patients on the site of the accident appeared to be appropriate to identify circulating DAMPs (Timmermans et al. 2016[312]). A transcriptomic commercially available test (Septicyte^®^) appears to be of interest to discriminate between sepsis and infection-negative systemic inflammation in critically ill patients (AUC ≤ 0.95) (McHugh et al. 2015[207]). A validation of this test was recently published (Balk et al. 2024[14]). However, in a meta-analysis on the use of transcriptomic for the diagnosis of sepsis which included a total of 117 studies and 17,469 patients, the differentiation between sepsis and patients with non-infectious systemic inflammatory response syndrome (SIRS) only led to an AUC = 0.84 (Loi et al. 2025[192]).

Among other biomarkers, miRNA have been suggested to be useful markers of infection and sepsis (Correia et al. 2017[73]; Zhang et al. 2019[360]). A meta-analysis was performed, enrolling 50 studies totaling 5225 sepsis patients and 4008 controls, involving 48 miRNAs. It was shown that total mixed miRNAs had a combined AUC of 0.86. The study revealed that certain miRNAs, specifically miR-155-5p, could be useful biomarkers for detecting sepsis (Zheng et al. 2023[365]).

Biomarkers have been also employed to further illustrate severity of sepsis. This has been shown a long time ago while measuring circulating cytokines such as IL-6 (Muñoz et al. 1991[218]), or IL-8 (Marty et al. 1994[203]), and was similarly observed with numerous plasma markers. Patients displaying a persistent elevated neutrophil to lymphocyte ratio endotype simultaneously exhibited heightened inflammation and pronounced immunosuppression (Restrepo et al. 2025[252]). Furthermore, biomarkers can be used as prognosis factors. For example, a study provided a robust prognostic model based on twenty-two differentially expressed immune-related genes which could predict 28-day mortality (Zheng et al. 2022[366]). Finally, biomarkers can be used to address the appropriateness of antibiotic therapy. It was recently reported that measurement of PCT reduces antibiotic duration safely compared with standard care (Dark et al. 2025[83]). Recently, in addition to analysis of blood samples, it has been proposed that urine-based metabolomics and proteomics profiles could be an useful tool for the diagnosis of sepsis (Bandyopadhyay et al. 2022[15]; Batra et al. 2023[18]). In patients with suspected lower respiratory tract infection, microorganisms could be identified in broncho-alveolar lavages, tracheal aspirates, or pleural fluids. Respiratory metagenomics provide benefits for antimicrobial treatment (Charalampous et al. 2024[60]).

Biomarkers are also needed to decipher between viral *versus* bacterial infections. Combining myxovirus resistance protein A and CRP led to a modest AUC = 0.69 (Iliopoulou et al. 2024[146]). In contrast the analyses of the expression of CD64 expression on neutrophils, and CD169 on monocytes allowed to distinguish the patients with a high AUC (0.93 and 0.97, respectively (Bourgoin et al. 2019[27]). A combined approach using three biomarkers (plasma proteins & cell-surface markers) has allowed to decipher between viral and bacterial infection with AUC ≥ 0.94 (Oved et al. 2015[228]; Velly et al. 2021[319]). Blood transcriptomic analyses have also allowed to discriminate between bacterial and viral infections in the emergency department with high AUC values (i.e. ≥ 0.93) (Ross et al. 2021[258]; Sampson et al. 2020[261]). A multi-omic approach using metabolomic, lipidomic, and proteomic analyses allowed to decipher between COVID-19 and bacterial sepsis-induced acute respiratory distress syndrome (ARDS) (Batra et al. 2022[19]). Integrating host transcriptional profiling and broad-range metagenomic pathogen detection from nucleic acid appears as a promising tool for viral sepsis diagnosis (Kalantar et al. 2022[154]).

## Sepsis: A Questionable Definition

**Figure 1:**
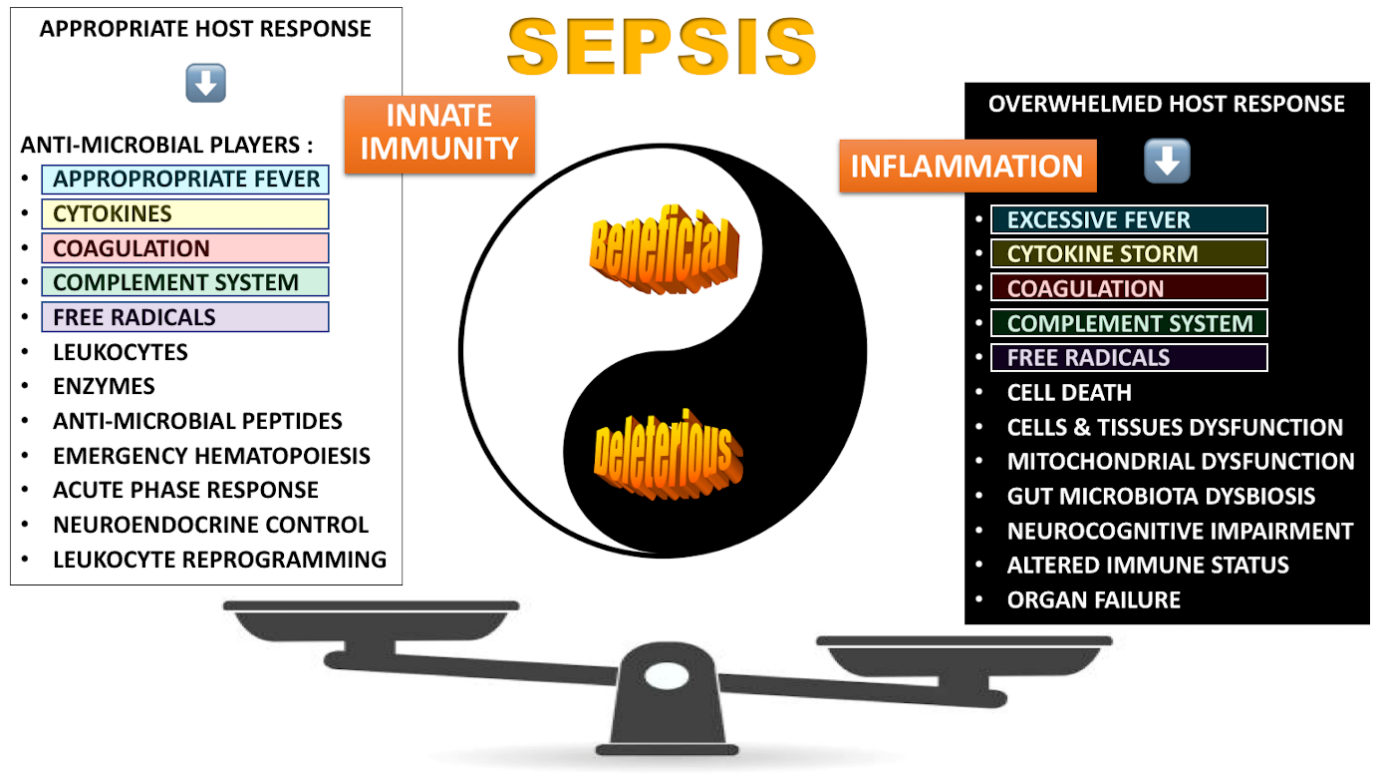
Graphical abstract. Sepsis results from a dysbalance between an appropriate immune response to an infectious insult and an overwhelmed response that ends in a global cellular and organ dysregulation. Of note, many players display a yin yang duality and contribute to both an appropriate immune response and a deleterious inflammatory response.

Since the initial paper offering the first consensual definition of “sepsis” associated with a systemic inflammatory response syndrome (SIRS) (Bone, 1992[24]), a huge number of papers, controversies and pro-con debates have been published, and discussed during many international meetings. In 2002, a new definition was offered (Levy et al. 2003[179]), considering sepsis, severe sepsis and septic shock to reflect different degree of severity, associated with any type of infection. Then, the term “severe sepsis” disappeared when a third definition of sepsis was proposed, which included the presence of two, or more organ system failures (Singer et al. 2016[279]). Despite this worldwide recognized definition (the paper has been cited more than 17,135 x by May 4^th^, 2025!), few authors raised concerns about it (Carneiro et al. 2017[43]; Sartelli et al. 2018[262]). The sepsis 3 new definition includes the assertion that sepsis represents a “dysregulated host response”. However, the word “dysregulated” might not be the most pertinent. Any healthy person, with a fully functional immune system can develop sepsis (figure 1(Fig. 1)). The very first and pertinent immune response will be aimed to fight the infection. Nature has selected through evolution the most appropriate response to an infectious insult, thus without any dysregulated process (Carlet, Submitted[38]). But, in the case of sepsis, by the end, an overwhelmed response has to be fully recognized, affecting in a proportional fashion both pro- and anti-inflammatory arms (Cavaillon et al. 2023[47]). The probable reason of this overwhelmed response is the synergy that exists between pathogen associated molecular patterns (PAMPs), bacterial toxins, cytokines, lipid mediators, free radicals, complement system, coagulation players, neuro-mediators, and damage associated molecular patterns (DAMPs) released by dead cells, etc… The respective role, of all these mediators, their synergy and their cross-talk remains to be further deciphered.

Despite the well-recognized sepsis 3 definition, there is still room for improvement as the definition does not provide a gold standard approach to identify cases of sepsis (Seymour et al. 2016[269]; Angus et al. 2016[7]). Of note, the new bedside clinical score termed quick Sepsis-related Organ Failure Assessment (qSOFA) was reported to have an inadequate sensitivity for early risk assessment (Giamarellos-Bourboulis et al. 2017[115]). Therefore, a complete SOFA score remains mandatory to assess the severity of sepsis patients.

## Epidemiology: A Global Concern

The incidence of sepsis varies among countries, whether the sepsis 2.0 or sepsis 3.0 definitions was applied. But a similar increase of the incidence was noted throughout the last decades. For example, in Spain in a study including 2,646,445 patients with sepsis, with 18.4 % mortality, the incidence of sepsis (events per 1000 population) increased from 3.30 (2000-2004) to 4.28 (2005-2009) to 4.45 (2010-2013) (p < 0.001) (Álvaro-Meca et al. 2018[5]). In Germany, the number of cases of sepsis rose by an average of 5.7 % per year, from 200,535 in 2007 to 279,530 in 2013, corresponding to an increase in the adjusted in-hospital incidence from 256 to 335 cases per 100 000 persons per year (Fleischmann et al. 2016[102]). In France, the sex-standardized and age-standardized incidence per 100 000 (95 % CI) increased from 357 in 2015 to 403 in 2019 and remained higher for males compared with females (Pandolfi et al. 2022[230]). In the USA, between 1979 and 2000, there was an annualized increase in the incidence of sepsis of 8.7 percent, from about 164,000 cases (82.7 per 100,000 population) to nearly 660,000 cases (240.4 per 100,000 population) (Martin et al. 2003[202]). The following years, the proportion of infection hospitalizations with a sepsis continue to increase from 7.5 % in 2003 to 11.5 % in 2009 (54 % increase) (Walkey et al. 2015[324]). In 2017, an estimated 48.9 million incident cases of sepsis were recorded world-wide and 11 million sepsis-related deaths were reported, representing 19.7 % of all global deaths, and mortality decreased by 52.8 % from 1990 to 2017 (Rudd et al. 2020[259]). Sepsis incidence and mortality varied substantially across regions, with the highest burden in sub-Saharan Africa, Oceania, South Asia, East Asia, and South East Asia. For example, in Africa, nine studies conducted in a hospital-wide setting showed a pooled prevalence and mortality of 17 % and 15 %, respectively. Five studies in the ICUs presented a pooled prevalence and mortality of 31 % and 46 %, respectively (Kiya et al. 2024[166]). In Japan, the analysis of 50,490,128 adult inpatients admitted between 2010 and 2017, revealed that the absolute number of sepsis and death in inpatients with sepsis significantly increased; however, the frequency of mortality rates and length of hospital stay of inpatients with sepsis significantly decreased (Imaeda et al. 2021[147]).

An estimated 16.5 million global deaths with sepsis was occurring in 1990. It decreased to 14.1 million deaths in 2019, before increasing to 21.4 million (20.3-22.4) deaths in 2021. In children younger than 5 years, deaths with sepsis decreased by more than 60 % over the past 31 years, from 7.69 million in 1990 to 3.14 million in 2019, and 2.68 million in 2021. In people 5 years and older, however, deaths with sepsis more than doubled over the study period, from 8.81 million in 1990, to 11.0 million in 2019, and 18.7 million in 2021, of which 7.89 million were due to COVID-19 (GBD 2021 Antimicrobial Resistance Collaborators[113]). The 2030 World Sepsis Declaration, a document adopted by delegates and supporter organizations of the World Sepsis Day event 2023 in Berlin aims to decrease by 2030, the incidence of sepsis from 677 episodes per 100,000 population per year to fewer than 500 episodes per 100,000 per year. 

## Host Response to Sepsis

The response of the host is extremely complex, and involves the activation of a huge number of networks, and the down-regulation of others. It is very difficult to summarize such a multi-factorial pathophysiology, and the authors of this article decided to present an up-dated overview of the data rendered available these last few years concerning those topics.

### Dysregulated cells 

#### Neutrophils

In humans, as well as in non-human primates, polymorphonuclear neutrophils (PMN) are the most abundant circulating leukocytes in contrast to rodents (mice and rats), rabbits, and pigs in which lymphocytes constitute the most profuse cell population within the blood stream. In all species, neutrophils are key players against infections, particularly thanks to their capacity to capture the pathogens by phagocytosis, as recognized in 1890 by Elie Metchnikoff who called them microphages (Metchnikoff, 1890[211]). More recently another means to seize bacteria was discovered by Zychlinsky's team (Brinkmann et al. 2004[29]) who reported the formation of neutrophil extracellular traps (NETs), a phenomenon called netosis which is associated with the death of the cells. Of interest, it was noticed that NET formation was higher after incubation of cells with plasma from either septic patients or septic mice, a phenomenon which was prevented by targeting the chemokine receptor CXCR1/2 (Alsabani et al. 2022[4]). It was reported that NETs, through activation of stimulators of interferon genes (STING), induce endothelial cell damage with abundant production of tissue factor, which magnified the coagulant cascade and lead to sepsis-induced acute lung injury (Zhu et al. 2021[369]). In addition to their capacity to favor coagulation, NETs are implicated in the pathophysiology of sepsis including the amplification of the inflammatory response, and are key players in the dysregulated host immune system (Retter et al. 2025[253]). Neutrophils are also equipped with enzymes and peptides that display microbicidal activity, and they contribute to the production of superoxide anion and nitric oxide. They expressed specific chemokine receptors that allow them to rapidly migrate towards infected or inflamed tissues. However, during sepsis, the migration abilities and antimicrobial functions of neutrophils are impaired (Zhou et al. 2022[367]). Similarly, it was reported an impaired capacity of PMNs from sepsis patients to produce cytokines such as interleukin-1β (IL-1β), IL-8 and IL-1 receptor antagonist (IL-1Ra) (McCall et al. 1993[205]; Marie et al. 1998[200]; Marie et al. 2000[201]). Initially believed as short-life cells with a half-life of less than 10 hours, they are now considered to display a lifespan of 5.4 days (Pillay et al. 2010[244]). During sepsis, length of life of neutrophils is increased as these cells display impaired CD24 expression (at both mRNA and protein levels), while CD24 ligation induces death through depolarization of the mitochondrial membrane in a manner dependent on caspase-3 and caspase-9 and reactive oxygen species (Parlato et al. 2014[232]). Neutrophils isolated from early deceased septic patients showed signs of hyperactivation (possibly due to excessive type-I IFN signaling), impaired functionality, recent exit from the cell cycle, and prolonged life-span (suggested by annexin A3 and vimentin protein levels) (Hortová-Kohoutková et al. 2021[134]).

In a murine model of lipopolysaccharide (LPS) induced acute lung injury (ALI), three subsets of neutrophils were identified within the lungs (circulatory, activated, and aged), and a cross-talk between neutrophils and other cells modulates neutrophil function. For example, mesenchymal stem cell treatment shifts the activated neutrophil phenotype into an aged neutrophil phenotype by upregulating the expression of CD24, reducing chemotaxis, reactive oxygen species (ROS) production, NADPH oxidation, and the secretion of granules (Feng et al. 2023[99]). Still in a murine model of sepsis, another subset of neutrophil has been identified, displaying immunosuppressive activity, expressing high levels of PD-L1 and suppressing the innate immune response by upregulating the proportion of Tregs (Qi et al. 2021[246]) and in human sepsis, a CD66b+ subset is enriched and inhibits proliferation and activation of CD4+ T cells (Kwok et al. 2023[170]). 

#### Myeloid derived suppressor cells and emergency hematopoiesis

Septic condition boosts the bone marrow to produce an increased number of immune cells needed to address the infectious process, a phenomenon known as emergency hematopoiesis. It includes the release of myeloid-derived suppressor cells (MDSCs). They are derived from bone marrow hematopoietic precursors due to the modified myelopoiesis consecutive to sustained production of inflammatory mediators and hematopoietic growth factors. MDSCs derived from the monocytic lineage (M-MDSCs) can be readily identified by the presence of CD14 and the decrease in HLA-DR molecules. On the other hand, MDSCs originating from the neutrophilic lineage (PMN-MDSCs) display a nonspecific immature phenotype, alongside their immunosuppressive functions. They are to be distinguished from immature neutrophils (Coudereau et al. 2024[74]). Interestingly, the high abundance of PMN-MDSCs and M-MDSCs within the bloodstream appears as a good predictor of mortality (Hollen et al. 2019[132]; Schrijver et al. 2024[264]). Of note, high levels of PMN-MDSCs were also present in long-term survivors many months after discharge, suggesting a possible role in sepsis-related complications (De Zuani et al. 2021[86]). MDSCs obtained at and beyond 14 days post-sepsis significantly suppress T lymphocyte proliferation and IL-2 production and express a specific pattern of miRNA (Hollen et al. 2019[132]). Epigenetic and transcriptomic signatures of emergency granulopoiesis in hematopoietic stem and progenitor cells and STAT3-mediated gene regulation is a hallmark of sepsis (Kwok et al. 2023[170]). MDSC expansion and its tissue-infiltration induce significant pathophysiology including lymphopenia, host immunosuppression and immune-paralysis that contribute to worsen patient outcomes (Malavika et al. 2022[197]). However, MDSCs obtained early or late after sepsis may have different properties. Adoptive transfer of early (day 3) MDSCs from septic mice into naive mice after cecal ligature and puncture (CLP) increased proinflammatory cytokine production, decreased peritoneal bacterial growth, and increased early mortality. Conversely, transfer of late (day 12) MDSCs from septic mice had the opposite effects. Early MDSCs express nitric oxide and proinflammatory cytokines, whereas late MDSCs express arginase activity and anti-inflammatory interleukin 10 (IL-10), and transforming growth factor-β (TGFβ) (Brudecki et al. 2012[31]).

#### Monocytes / macrophages

Monocytes / macrophages are both the culprits of sepsis-associated tissue damages and the victims of the overzealous immune response (Gao et al. 2023[109]). As main producers of pro- and anti-inflammatory cytokines, they contribute to both the systemic inflammatory response syndrome and the compensatory anti-inflammatory response syndrome (Adib-Conquy & Cavaillon 2009[2]). Macrophages come as two flavors, the M1 macrophages catabolize arginine via inducible nitric oxide synthase and contribute to the fight against the insult, and M2 catabolize arginine via arginase and contribute to cell proliferation and tissue repair (Mills et al. 2000[212]). This is illustrated by a study which showed that M2 macrophages could promote endothelial cells proliferation in sepsis-induced acute lung injury through secretion of anti-inflammatory cytokines and growth factors (Shen et al. 2019[275]). An imbalance between M1 and M2 macrophages occurs during the occurrence and development of sepsis. Signaling molecules, transcription factors, epigenetic modifications, and metabolic reprogramming contribute to macrophage polarization which is time (early *versus* late phase) and site-environment dependent (Wang & Wang 2023[331]). 

The yin yang contribution of macrophages is illustrated by their deleterious contribution to sepsis-associated acute lung injury (Dang et al. 2022[82]), acute kidney injury (He et al. 2023[127]) or encephalopathy (Yan et al. 2022[348]). While Macrophages can promote inflammation and tissue injury, they also display beneficial properties by favoring resolution of inflammation. For example, cardiac-resident CD163+ TREM2hi macrophages protect the septic heart by maintaining cardiomyocyte homeostasis (Zhang et al. 2023[356]). On another hand, Ly6Chigh monocytes protect against kidney damage during sepsis via a CX3CR1-dependent adhesion mechanism (Chousterman et al. 2016[70]).

Mesenchymal stem cells can protect against sepsis by inhibiting M1 polarization (Liang et al. 2019[188]). Among the macrophage-dependent protective mechanisms recently identified let's mention the role played by the CD169+ macrophages which control inflammatory responses via interleukin-10 (Yeung et al. 2023[351]), and the Krüppel-like transcription factor-14 (KLF14) of which the expression is upregulated in septic patients and which decreases glycolysis and the secretion of inflammatory cytokines by macrophages, by inhibiting the transcription of the key glycolytic enzyme, hexokinase 2 (HK2) (Yuan et al. 2022[353]).

#### Cell death

**Figure 2:**
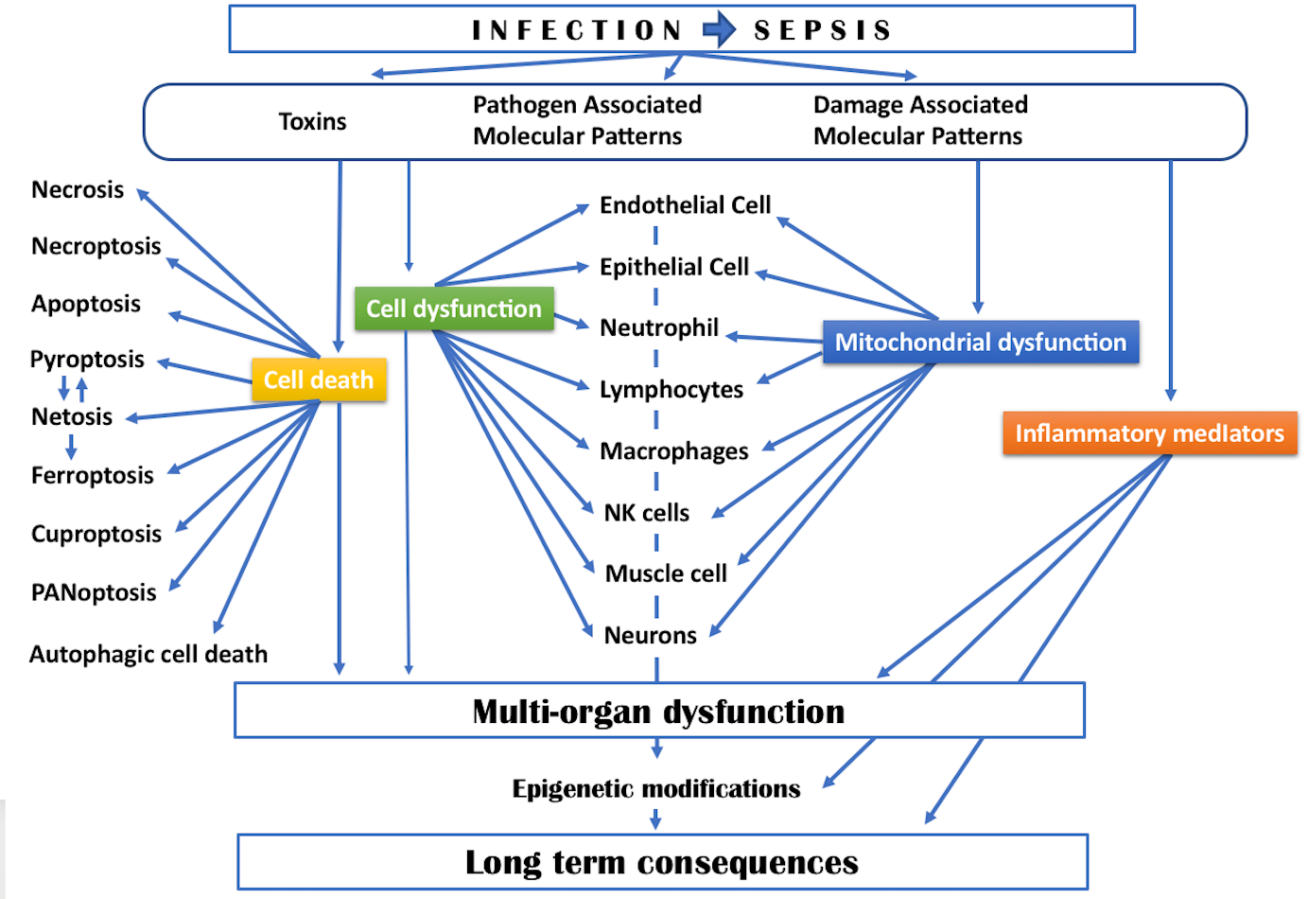
Figure 2. Sepsis induces numerous alterations at the cellular level that can either lead to cell death or cell dysfunction, associated with intracellular dysfunction, such as that of the mitochondria. Different types of cell deaths have been reported in sepsis. These events together with the overwhelming production of inflammatory mediators contribute to organ dysfunction. Associated epigenetic modifications result in a regulation of the inflammatory response, and can allow a more appropriate response to a second insult, or result in negative long-term consequences.

Programmed cell death or apoptosis has been recognized a long time ago to occur in sepsis, to be responsible of lymphopenia (Ayala & Chaudry 1996[11]; Hotchkiss et al. 2003[138]) and to contribute to an alteration of the immune status (Hotchkiss & Nicholson 2006[137]). But programmed cell death can also occur in parenchymal cells ending in organ failure (Wang et al. 2025[330]). As illustrated in figure 2(Fig. 2), many other cell death processes have been identified and characterized in sepsis. 

Necrosis of skin and soft-tissue is a devastating consequence of some specific toxin-producing virulent bacteria. Necroptosis represents a regulated version of the necrotic cell death pathway induced by the action of tumor necrosis factor (TNF) or Fas ligand. Other means of induction of necroptosis have been described such as that epithelial cells by a macrophage-derived exosomal aminopeptidase N (APN/CD13) which aggravates sepsis-induced acute lung injury. Exosomal APN/CD13 regulated necroptosis of lung epithelial cells by binding to the cell surface receptor TLR4 to induce ROS generation, mitochondrial dysfunction and NF-κB activation (Gong et al. 2022[119]).

We have earlier eluded to netosis which can be induced by bacteria, fungi, parasites and viruses, microbial derived products (LPS, N-formylmethionyl-leucyl-phenylalanine [fMLP]) and also by endogenous mediators (IL-8, TNF, CXCL2, platelet activating factor [PAF], H2O2). 

Inflammasome which contributes to the production of IL-1β and gasdermin D-dependent cell death or pyroptosis induces neutrophils to extrude antimicrobial NETs (Chen KW et al. 2018[62]; Chen et al. 2021[65]). For other authors, if NETs can be formed by viable neutrophils after inflammasome activation, this function does not require gasdermin D (Stojkov et al. 2023[293]). In an opposite fashion, it has been reported that NETs can favor macrophage pyroptosis in sepsis (Chen L. et al. 2018[63]). Furthermore, netosis has been reported to activate ferroptosis in alveolar epithelial cells, contributing to the pathology of sepsis-associated acute lung injury (Zhang H. et al. 2022[355]). Ferroptosis is a cell death process, associated with an accumulation of lipid peroxides which is iron-dependent, and thus can be inhibited by iron chelators (Li et al. 2020[181]). The death of renal tubular epithelial cells in a sepsis-associated acute kidney injury has been associated with the presence of ferroptosis in these cells (Xiao et al. 2024[343]). It involves the rhythm gene nuclear factor interleukin-3 (NFIL3) which may act as a transcriptional activator of ACSL4, the gene coding for the long-chain-fatty-acid-CoA ligase 4, an enzyme that catalyzes the conversion of long-chain fatty acids to their acyl-CoA active form for both synthesis of cellular lipids and degradation via beta-oxidation. The stimulator of interferon response cGAMP interactor 1 (STING1) encodes a five transmembrane protein that functions as a major regulator of the innate immune response to viral and bacterial infections. The nuclear receptor coactivator 4 (NCOA4) acts as a regulator of DNA replication origins that helps to prevent inappropriate DNA synthesis and replication stress. It has been suggested that STING regulates macrophage ferroptosis and promotes sepsis-induced intestinal injury via its interaction with NCOA4 (Wu et al. 2022[337]). In macrophages, 4-octyl itaconate (4-OI), a metabolite produced during inflammatory macrophage activation, inhibits ferroptosis induced by LPS and alleviated sepsis-induced ALI. 4-OI inhibits the nuclear factor erythroid 2-related factor 2 (Nrf2) degradation and promotes the transcription of target genes, including those coding for cystine/glutamate transporter (SLC7A11), glutathione peroxidase 4 (GPX4), and* glutamate-cysteine ligase* (GCLM) (He R. et al. 2022[128]). Cuproptosis is a newly described form of cell death. It is triggered by targeted accumulation of Cu in mitochondria that drives lipoylated tricarboxylic acid (TCA) cycle enzyme aggregation via direct Cu binding resulting in proteotoxic stress (Tsvetkov et al. 2022[314]). It has been recently reported that cuproptosis-related genes are either significantly up-regulated or down regulated in peripheral blood lymphocytes of septic patients as compared to healthy controls (Zhao et al. 2024[363]). The authors considered that these genes exhibit potential diagnostic efficacy in septic shock, and could be potential biomarkers for the diagnosis of septic shock. Likewise, a risk score for sepsis identification has been established based on ten key pyroptosis-related genes, four of which also have potential value for the prognosis of sepsis (Sun et al. 2023[300]). In a murine model of sepsis-induced acute kidney injury, up-regulation of macrophage migration inhibitory factor (MIF) contribute to renal damage aggravating NLRP3 inflammasome mediated cell pyroptosis (Li et al. 2023[182]).

The interaction between pyroptosis, apoptosis and necroptosis is involved in PANoptosis. It is initiated by innate immune sensors, pattern-recognition receptors (PRRs) in response to PAMPs, DAMPs, and cytokines, and driven by caspases and receptor-interacting protein kinases (RIPKs) (Malireddi et al. 2019[198]). In addition, S100A8/A9, which is mainly released by the neutrophils during sepsis, disrupts mitochondrial homeostasis and induces PANoptosis in endothelial cells (Wang et al. 2024[328]). In COVID-19, endothelial cell death by necroptosis was reported to induce complement-dependent red blood cell haemolysis. Deposition of haemolysed RBC membranes at sites of endothelial cell death favor microvascular obstruction (Wu et al. 2025[338]).

PANoptosis‐related genes effectively distinguished septic patients from those septic patients who developed ARDS: N-Myc Downstream Regulated 1 (NDRG1) showed a positive correlation with activated dendritic cells, whereas DEAD-box helicase family member (DDX3X), Protein tyrosine phosphatase, receptor type, C (PTPRC), and TNF Superfamily Member 8 (TNFSF8) were positively associated with neutrophils and negatively correlated with CD56bright NK cells (Lu et al. 2025[194]).

Autophagy is a protective process by which cells degrade and recycle proteins and organelles to maintain intracellular homeostasis. But disruption of autophagy mechanisms or excessive autophagic flux usually leads to cell death (Liu et al. 2023[191]), especially ferroptosis (Li et al. 2023[182]). The autophagic cell death, like necroptosis, pyroptosis, and ferroptosis, leads to the release of inflammatory cytokines and DAMPs, and amplify inflammation (Iba et al. 2024[144]). Nevertheless, number of sepsis-related studies have shown that autophagy plays a protective role in multiple organ injuries, partly by clearing pathogens, regulating inflammation and metabolism, inhibiting apoptosis and suppressing immune reactions (Feng et al. 2019[100]). Accordingly, multiple drugs and measures have been reportedly beneficial for septic challenge by inducing autophagy process. Therefore, autophagy might be an effective target for reversing immunosuppression favored by sepsis (Ren et al. 2017[251]). Among 80 differential autophagy-related genes, seven were identified as hub genes and diagnostic biomarker groups for human sepsis (Di et al. 2023[90]).

### Cellular dysregulation 

If we considered that sepsis is not consecutive to a pre-existing dysregulation of the host response, in contrast the excessive host response observed during sepsis induces cellular and organ dysregulation.

#### Altered Immune status

While we consider that at the initiation of the response to the insult consecutive to a microbial invasion, Nature offers the appropriate response, we also think that the concomitant anti-inflammatory response is the appropriate response to counteract the inflammatory response. Indeed, there is a need of a proportional anti-inflammatory response to address an overzealous host response. The reprogramming of circulating immune cells is aimed to avoid the perpetuation of the inflammatory response and to dampen the capacity of the immune cells to release an excess of inflammatory cytokines. But the excessive counter-regulation will lead to the so called “compensatory anti-inflammatory response syndrome” (CARS), which itself lead to an altered immune status and an increased sensitivity to subsequent new infections (Adib-Conquy & Cavaillon, 2009[2]). During sepsis, circulating monocytes display altered functionality such as a decreased capacity to release cytokines, including IL-1β, upon stimulation (Muñoz et al. 1991[217]) and display a reduced expression of HLA-DR (Monneret et al. 2004[213]). IL-1β is produced following the inflammasome molecular platform to which belongs the “apoptosis-associated speck-like protein containing a CARD” (ASC) molecule. Following activation of the inflammasome, ASC assembles into a large protein complex called “speck”. Sepsis patients exhibited reduced ASC speck-positive monocytes. Of note, *ex vivo* activation of the NLRP3 inflammasome in circulating monocytes and neutrophils from patients with bacterial and viral sepsis is observed in association with increased mortality (Coudereau et al. 2023[75]). Toll-Interleukin Receptor (TIR) domain-containing adapter molecule 2 (TICAM-2) is involved in Toll receptor signaling. Using a murine cecal slurry injection sepsis model, and studying blood samples collected from a sepsis patient, it appears that TICAM-2 contribute to a reprogrammed profile of monocytes (Caldwell et al. 2025[36]). Reprogrammed monocytes of sepsis patients display an altered DNA methylation throughout the genome, including genes implicated in immune dysregulation during bacterial and viral sepsis. These changes are recapitulated in septic mice induced by cecal slurry injection (Caldwell et al. 2024[37]). In a CLP model, a long-term deployment of functionally impaired inflammatory monocytes to the vasculature of non-lymphoid organs was observed during the recovery phase, with limited activation capacity, impaired phagocytic activity and failure to favor lung recovery and protection against secondary infections (Baudesson de Chanvile et al. 2020[20]). In contrast, sepsis-surviving mice showed enhanced resistance to* Listeria *infection. Sepsis survival was associated with an induced lipid metabolic reprogramming in myeloid cells, contributing to enhanced immunity against the intracellular pathogen (Watanabe et al. 2025[333]).

Among the cellular cross-talks which contribute to the altered immune status of circulating cells, specific PD-L1+ CD44+ B220Low CD138+ IgM+ regulatory plasma cells have been identified in the spleen of septic mice. They regulate the *ex vivo *proliferation and IFNɣ secretion induced by stimulation of T splenocytes. This effect was shown to be mediated both by cell-cell contact through increased PD-L1 expression on plasma cells and by production of a soluble factor. A similar PD-L1+ regulatory plasma cells in septic patients has been observed in the blood of septic patients (Gossez et al. 2025[120]).

The reduced capacity to release cytokine recalls the process of endotoxin tolerance (Cavaillon & Adib-Conquy, 2006[51]). Indeed, two types of leukocyte reprogramming exist. One is aimed to dampen the overzealous activity of these cells, the other one, called innate immune memory or trained innate immunity, allows faster and stronger immune response to a secondary challenge (Wang et al. 2024[328]). Initially described to be observed with monocytes (Kleinnijenhuis et al. 2012[167]; Ifrim et al. 2014[145]), it was shown that agonists that favor trained immunity such as β-glucan or BCG could reverse LPS-induced tolerance (Novakovic et al. 2016[223]). *In vitro* induction of trained immunity by β-glucan on monocytes from sepsis patients enhances their metabolic capacity, and increased their cytokine production similarly to that obtained with monocytes from healthy subjects (Gill et al. 2022[116]). Of note, while IL-4 has been known for a long time to inhibit the production of inflammatory cytokines (Weiss et al. 1989[335]), IL-4 has been shown to induce a long-lasting innate immune memory / trained immunity. Furthermore, in mice and non-human primates, an apoA1-IL-4-embedding nanoparticles target was demonstrated to resolved immunoparalysis in mice with LPS-induced hyperinflammation, as well as in *ex vivo* human sepsis models and in experimental endotoxemia (Schrijver et al. 2023[263]).

Most interestingly, a chemokine network released from sepsis-trained resident macrophages triggers tissue anti-tumoral residency of T cells via CCR2 and CXCR6 stimulations and promotes the occurrence of mechanisms responsible for a decreased risk of *de novo* tumor development (Broquet et al. 2024[30]). In contrast, innate immune memory mediates increased susceptibility to Alzheimer's disease-like pathology in sepsis surviving mice (De Souza et al. 2021[85]). Other immune cells can undergo the process of trained innate immunity. For example, it has been observed that sepsis induces innate immune memory in stressed granulocytes which can boost fatal inflammatory responses to secondary infections, knowing that stressed granulocytes specifically target lungs by the CXCL2-CXCR2 axis. Chromatin remodeling of the TLR4 pathway and metabolic shift have been reported in these cells (Wang et al. 2023[326]). Similarly, NK cell memory develops after endotoxemia in a histone methylation-dependent manner, ensuring a heightened response to secondary stimulation (Rasid et al. 2019[248]).

As addressed in other reviews, the altered immune status during sepsis is also the consequence of dysregulated functions of lymphocytes (Nedel et al. 2025[219]), dendritic cells (Zheng et al. 2024[364]), and neutrophils (Zhang et al. 2025[357]). Among the molecular culprits, let's mention the programmed cell death 1 (PD-1) receptor and its ligand (PD-L1) (Patera et al. 2016[235]), the cytotoxic T lymphocyte antigen 4 (CTLA-4) (Inoue et al. 2011[149]), the T cell Ig and ITIM domain (TIGIT) coinhibitory receptor (Zhang et al. 2021[359]), and the T cell immunoglobulin and mucin domain 3 (Tim-3) surface molecule (Luo et al. 2024[195]). These surface inhibitory immune checkpoint receptors and ligands appear as potential therapeutic targets to counteract the immunosuppressed profile of sepsis patients (McBride et al. 2021[204]).

#### Endothelial dysfunction

Due to their strategic localization at the interface between the bloodstream and tissues, endothelial cells play an important role in propagating the inflammatory process throughout the body. Thus, their integrity is key to maintain homeostasis, vascular barrier function, coagulation pathways, leukocyte adhesion, and vasomotor tone. During sepsis, their activation favors tissue factor expression and coagulation, and leukocyte transmigration towards tissue. Their alteration will be associated with a loss of endothelium integrity, vascular leakage and organ dysfunction (de la Fuente et al. 2024[84]). Endothelial cells can become activated by inflammatory cytokines (e.g. IL-1, IL-18, TNF, CCL2…), free radicals (NO, ROS), thrombin, bradykinin, histamine, CRP, and vascular endothelial growth factor (VEGF) (He et al. 2024[126]). More recently the NETs formation was added to the list of activators (Zhu et al. 2021[369]; Zhang et al. 2023[356]). Interestingly, *in vitro* exposure of endothelial cells (Human umbilical vein endothelial cells, HUVEC) to patients' sera, led to their activation in correlation with sepsis severity, including significantly increased expression of adhesion receptors (ICAM-1, VCAM-1), the release to the extracellular matrix of von Willebrand factor, augmented thrombogenicity toward platelets, and increased phosphorylation of intracellular p38MAPK (Fernandez et al. 2021[101]). Furthermore, sepsis plasma induced areas of endothelial cell contraction, loss of cellular coverage, and alteration of the barrier function (Liang et al. 2024[187]). Endothelial markers (e.g. E-selectin, P-selectin, ICAM-1, VCAM-1…) released to the blood have been proposed as diagnostic biomarkers for acute infection and sepsis. Despite promising results in small studies, a recent study on 312 adult patients in emergency department revealed a limited diagnostic utility (Galtung et al. 2025[107]). Numerous studies illustrate that strategies aimed to prevent endothelial dysfunction could result in improving the outcomes of sepsis patients (Girardis et al. 2024[117]).

#### Neurocognitive Dysfunction

More than half of sepsis survivors have long-term cognitive impairment. Cerebrovascular damage, metabolic disorders, and brain inflammation are hallmarks of sepsis and precede cognitive impairment (Annane & Sharshar, 2015[9]). Nearly two months after CLP, sepsis survivor mice demonstrate cognitive changes in the absence of neuronal loss or changes in synaptic density in the hippocampus. An infiltration of monocytes and neutrophils into the central nervous system at least two weeks after sepsis in a CCR2 independent manner was noticed. The cellular inflammation is accompanied by long-term expression of pro-inflammatory cytokine and chemokine genes, including TNF and CCR2 ligands, in whole brain homogenates (Singer et al. 2016[279]). 

Investigating neurocognitive sequelae in sepsis patient survivors, a reduction in working memory storage capacity, and a crucial cognitive mechanism were observed resulting in cognitive deficits. These patients are unable to maintain the same quantity of information at a given moment as their healthy counterparts. This diminished capacity in working memory storage significantly contributes to the lower performance observed in standard neuropsychological tasks assessing attention and working memory (Kattlun et al. 2024[160]).

Several mechanisms have been proposed, including blood-brain barrier disruption, neuroinflammation, neurotransmitter dysfunction, microglial activation and neuronal loss (Li Y et al. 2022[186]). Behavioral task-associated positron emission tomography (beta-PET) identifies a biologically defined network encoding contextual threat memory and its uncoupling in a mouse model of long sepsis (Strohl et al. 2024[295]). Among the new players identified in neurocognitive dysfunction, let's mention the P2X7 receptor, a ligand-gated ion channel that is activated by extracellular adenosine triphosphate (ATP) released by stressed cells and abundantly expressed in the brain. Both wild type and P2X7−/− sepsis-surviving mice showed memory impairment 13 days after CLP, and both groups of animals presented increased acetylcholinesterase (AChE) activity in the hippocampus and cerebral cortex. But, the absence of P2X7 partly prevented this increase in the cerebral cortex. Likewise, P2X7 absence decreased ionized calcium-binding protein 1 and glial fibrillary acidic protein (GFAP) upregulation in the cerebral cortex of sepsis-surviving animals. Accordingly, the P2X7 receptor contributes to chronic neurodegenerative and neuroinflammatory diseases (Alves et al. 2023[6]). Mast cells are another new player recently identified. Mast cells were found overactivated in the hippocampus of CLP induced sepsis-associated encephalopathy mice. Cromolyn, a substance which prevents release of allergic mediators, including histamine, intracerebroventricularly injected substantially inhibited the mast cells activation and neuroinflammation responses, ameliorated blood brain barrier impairment, improved the survival rate and alleviated cognitive dysfunction in septic mice. *In vitro* experiments revealed that mast cells activation increased the sensitivity of brain microvascular endothelial cells against to LPS challenge. Furthermore, the histamine 1 receptor (H1R) could mediate blood brain barrier impairment and cognitive responses by modulating the TLR2/4-MAPK signaling pathway (Yue et al. 2023[354]). A sepsis model of CLP coupled with a daily chronic stress (animals being placed into a restraint holder for 2 h daily for 7 days) used to generate robust systemic inflammation, allowed to provide evidence that sepsis influences brain immunity and brain connectivity in a sex-dependent manner. Both male and female mice, exhibited a comparatively robust intracerebral glial proliferation relative to their healthy counterparts. But analysis by functional magnetic resonance imaging (fMRI) reveals that intra-network connectivity strength in the striatum preferentially increased in post-septic males but remained near baseline in post-septic female mice. Additionally, the female mice showed reduced network connectivity alterations in the projections from periaqueductal gray to the superior colliculus and between the anterior cingulate cortex and the striatum (Vo et al. 2025[322]).

#### Mitochondrial dysfunction

Mitochondria generate most of the cellular energy, dependent on the generation of ATP, involving the electron transport chain. Nitric oxide and its metabolites, produced in considerable excess in patients with sepsis, can affect oxidative phosphorylation by inhibiting several of its component respiratory enzymes. Mitochondrial dysfunction resulting in bioenergetic failure may be an important factor in the pathophysiology of sepsis-associated multiorgan failure. However, a reasonable argument proposed by Mervyn Singer (Brealey & Singer, 2003[28]) can be made: the reduction in energy supply could represent an adaptive response to ongoing inflammation, resulting in a cellular shutdown analogous to hibernation that allows eventual restoration of organ function and long-term survival in patients. In agreement with this concept is the observation that LPS tolerance observed after sepsis protected septic animals from mitochondrial dysfunction, favoring mitochondrial biogenesis and preserving mitochondria respiration and respiratory complex I activity (Silva et al. 2024[278]). However, abnormal mitochondrial ultrastructure, impaired respiration and electron transport chain activities, and persistent protein oxidative damage found in the muscle of sepsis survivors are associated with chronic muscle weakness developed by these patients for at least one month, even after recovery of muscle mass (Owen et al. 2019[229]). 

ROS production within mitochondria can lead to oxidative damage to mitochondrial proteins, membranes, and mitochondrial DNA. Mitochondrial oxidative damage leads to the release of cytochrome c into the cytosol resulting in apoptosis (Galley et al. 2011[106]). Thus, it has been suggested, that antioxidant therapy might be useful. A recent meta-analysis reports that high-dose vitamin C, and the combination of vitamin C with vitamin B1 emerge as promising interventions, exhibiting the potential to reduce 28-day mortality and SOFA scores. Vitamin E, selenium, and *N*-acetylcysteine are deemed ineffective (Pham et al 2024[240]).

Mitophagy is the selective degradation of mitochondria through autophagy. This process occurs particularly in mitochondria that are defective due to damage or stress. It was reported that the inhibition of mitophagy triggered classical macrophage activation in a mitochondrial ROS-dependent manner, favoring bactericidal clearance, and improving the outcome of sepsis (Patoli et al. 2020[236]). However, a study reveals that a high degree of mitophagy may predict a good outcome of sepsis, and* Prohibitin 1, *a chaperone for respiratory chain proteins, is a key NLRP3 inflammasome regulator via mitophagy in sepsis (Chen et al. 2023[61]). Indeed, mitophagy is considered to be beneficial for sepsis by eliminating disabled mitochondria and maintaining homeostasis to protect against organ failure (Zhu et al. 2021[368]).

### Mediators

#### Old and new players

During the COVID-19 crisis, old players described in bacterial sepsis have been “rediscovered” as players of this viral sepsis. For example, this was the case of the anaphylatoxin C5a (Carvelli et al. 2020[45]), described 20 years earlier to contribute to sepsis and to be a putative target for therapies (Czermak et al. 1999[77]). Among the old player, there is also the pancreatic stone protein (PSP) which has been very much used as a biomarker. Some of its bio-activities have been recalled in a recent review (Ventura & Tissière, 2024[320]). PSP, is a C-type lectin protein secreted by the pancreas in response to stress such as sepsis. Animal studies have shown that PSP injection aggravates sepsis. In humans, studies have shown that PSP activates PMNs, favors neutrophil infiltration and microcirculatory failure, aggravates multiple organ dysfunction syndrome, and circulating levels correlate with poor outcome (Hu et al. 2023[140]).

Another old player is the high-mobility group box 1 (HMGB1) which has celebrated its 50^th^ years anniversary (Tang et al. 2023[308]). Initial discovery as a structural protein of chromatin, HMGB1 is now known to regulate diverse biological processes. HMGB-1 is a DAMP released as late mediator during sepsis, of which levels correlate with poor outcome, as it displays deleterious effects. Accordingly, it has been proposed to be an interesting target to improve outcome (Stevens et al. 2017[292]).

Sphingosine 1-phosphate (S1P) is another molecule which has been reported as an interesting biomarker as its serum levels in human sepsis are dramatically decreased and inversely associated with disease severity (Winkler et al. 2015[336]). It binds to carrier molecules like serum albumin and high-density lipoproteins (HDL) which contribute to the regulation of S1P effects. Lower complex formation of S1P with HDL in septic shock patients correlates with the need for mechanical ventilation and mortality (Seidita et al. 2024[267]). S1P is more abundant in intestinal tissues as compared to other tissues; S1P binds to five receptors and transmits different intracellular signals depending on the G protein-coupled G subunit. It exerts anti-inflammatory effects, promotes immune cell trafficking, and protects the intestinal barrier by maintaining the integrity of the intestinal intraepithelial vascular endothelial cells barrier (Sun et al. 2023[297]).

New interests have focused on angiopoietin-like 4, a serum hormone directly involved in regulating lipid metabolism. In mice, the highest mRNA expression levels of angiopoietin-like 4 are found in white and brown adipose tissue, and in humans it is particularly expressed in the liver. Recently, angiopoietin-like 4 has been identified as a key factor produced by the brain endothelium that preserves blood-brain barrier integrity during bacterial sepsis. Treatment with recombinant angiopoietin-like 4 reduced vascular leakage, organ failure and death in mouse models of lethal sepsis and *N. meningitidis* infection. Protection was conferred by a previously uncharacterized domain of angiopoietin-like 4, through binding to the heparan proteoglycan, syndecan-4 (Ziveri et al. 2024[371]). In total opposite, angiopoietin-like 4 silencing ameliorated sepsis-induced acute lung injury in mice, associated with a repressed M1 macrophage polarization and macrophage pyroptosis, and hindered LPS-induced activation of the NF-κB pathway in macrophages (Sun et al. 2024[296]). Further investigations are needed to decipher whether angiopoietin-like 4 is a beneficial, a deleterious factor, or both in a yin yang process.

Among the players of sepsis recently discovered is the phospholipase PLA2G5. PLA2G5 was identified by Nicolas Chevrier's team using whole-mouse spatial profiling to generate body-wide maps of systemic inflammation in a mouse model of endotoxemia. They demonstrated that the phospholipase PLA2G5 was a harmful factor that causes multi-tissue damage and death during endotoxemia and bacterial sepsis. Mechanistically, they found that bloodborne PLA2G5 leads to intravascular hemolysis through its lipolytic activity on red blood cell membranes. In humans with bacterial, viral, or fungal sepsis, the plasma levels of PLA2G5 are elevated and predictive of disease severity, suggesting that sepsis corrupts PLA2G5 into becoming a systemic self-venom which is toxic for red blood cells. *Pla2g5* gene is expressed in mouse gut at steady state and in cells of the human gastrointestinal tract, including in the stomach and colon. However, it remains unknown whether PLA2G5 is up-regulated in gut cells during human sepsis as they observed in mice. PLA2G5 antibodies protect against lethal LPS injection and PLA2G5 deficient animals survived LPS induced lethality (Takahama et al. submitted 304). Hemolysis is frequently observed among sepsis patients and its incidence is significantly higher among those with septic shock and those receiving extracorporeal membrane oxygenation (ECMO) therapy, particularly when associated with continuous renal replacement therapy. Haptoglobin and hemopexin serve as scavenger proteins, binding free hemoglobin and free heme to mitigate their toxic effects and facilitate their clearance from circulation. Their depletion in plasma is a strong indicator of ongoing hemolysis (Bąkowski et al. 2025[13]).

#### The current knowledge on the role of cytokines in sepsis

As mentioned above, the investigation on COVID-19 led to publications in high impact journals of information known for decades. For example, the inflammatory and deleterious synergy between TNF and IFNγ described in 1992 (Doherty et al. 1992[92]) was rediscovered during the COVID-19 crisis (Karki et al. 2021[158]). The concept of cytokine storm in sepsis has been widely used since it has been coined (Tyburski et al. 2001[315]). It reflects the overwhelmed immune response mentioned earlier (Cavaillon et al. 1992[57]). While the concept has been inappropriately employed to qualify the measurable circulating cytokines observed during severe COVID-19 (Osuchowski et al. 2021[227]), the concept could be employed when considering the events occurring within the lungs (Xiong et al. 2020[345]). The possibility to define drugs that would globally limit the cytokine storm and restore intracellular homeostasis could demonstrate therapeutic potential (Yan et al. 2023[347]). These approaches emerge after decades of investigations which were aimed to target one given cytokine using inappropriate animal models poorly sensitive to infection and LPS such as rodents and baboons (Cavaillon et al. 2020[58]). For example, the famous key publication in which TNF was neutralized in a baboon model of bacteraemia was achieved with the injection of 0.7 x 10^10^ *E. coli */ kg body (Tracey et al. 1987[313]). Knowing that the highest concentration of *E. coli* in feces is around 10^8^, the bacterial load used in this study would be equivalent to 10 kg of feces (Kassasseya et al. 2024[159]). Of course, many studies have demonstrated that TNF does play a role in the lethal effect of sepsis. In a very elegant recent investigation, it was further reported that TNF-induced lethality does not occur in mice with a Paneth cells-specific deletion in the TNF receptor, P55. In Paneth cells TNF stimulates an interferon signature and ablates the steady-state unfolded protein response (UPR). This UPR deficiency causes a significant reduction in antimicrobial peptide production, and Paneth cells antimicrobial activity, causing bacterial translocation to organs and subsequent polymicrobial sepsis, organ failure, and death (Wallaeys et al. 2024[325]).

As initially referred to, synergy is a master concept to understand the contribution of cytokines in sepsis. For example, interleukin-3 enhances the release of cytokines produced by macrophages *in vitro* upon LPS activation (Cohen et al. 1991[72]). In agreement, it was reported that IL-3 induces emergency myelopoiesis, potentiates inflammation in sepsis and fuels a cytokine storm while IL-3 deficient mice were protected against sepsis, and in humans with sepsis, high plasma IL-3 levels are associated with high mortality (Weber et al. 2015[334]). In contrast, illustrating the yin yang aspects of cytokines, it was reported that IL-3-deficient septic mice were more susceptible to pulmonary herpes simplex virus infection and exhibited higher pulmonary inflammation than control mice. Mechanistically, IL-3 increases innate antiviral immunity by promoting the recruitment of circulating plasmacytoid dendritic cells into the airways and by enhancing their capacity to activate T cell. Interestingly, the ability of IL-3 to improve adaptive immunity was confirmed in patients with SARS-CoV-2 infections (Bénard et al. 2023[21]).

The ambiguity on the role of the cytokines during inflammation and sepsis is also observed with the so-called anti-inflammatory cytokines. While some years ago we reported the ambivalent role of IL-10 (Petit-Bertron et al. 2005[239]), similar recent results have been obtained with transforming growth factor β (TGFβ) which does not behave only as an anti-inflammatory cytokine that down-regulate T-cells. Elevated levels of circulating TGFβ have been reported in sepsis patients (Marie et al. 1996[199]). Epigenetic changes induced by TGFβ, such as increased Smad3 activation and reduced proinflammatory transcription factor motif enrichment, contribute to the anti-inflammatory profile of macrophages. Despite its conventional anti-inflammatory role, TGFβ-treated macrophages exhibit a distinct phenotype marked by heightened glycolysis, suppressed proinflammatory cytokines, and increased coagulation factor expression, ending to a decrease survival in mouse models of sepsis. TGFβ reprograms macrophages during inflammation, creating a macrophage that metabolically and functionally differs from traditional M1 or M2 phenotyping (Gauthier et al. 2023[112]; Oliver et al. 2024[225]). TGFβ is responsible of a functional impairment in T cell reactivity, accompanied by a reduced antigen-presentation capability of monocytes. it has been found that the multisystem inflammatory syndrome which can occur in SARS-CoV-2 infected children was associated with an impaired T cell cytotoxicity triggered by TGFβ overproduction. As a consequence, an Epstein Barr virus (EBV) reactivation occurs, and subsequently a hyperinflammation was observed (Goetzke et al. 2025[118]).

Transforming growth factor beta induced (TGFBI) gene expression is regulated by TGFβ. Interestingly, its reduced expression during sepsis correlates with poor prognosis and rapid disease progression. TGFBI expression was significantly higher in M2-like macrophages, and was found to inhibit LPS-induced polarization and phagocytosis in M1-like macrophages, thereby playing a role in preventing the onset of inflammation. The protein-protein interaction network revealed the top ten genes that interact with TGFBI, showing significant involvement in the regulation of the actin cytoskeleton, extracellular matrix-receptor interactions, and phagosomes (Shi et al. 2024[276]).

The responses to IL-1β, IL-6, and TNF are significantly overlapping, highlighting a redundant cytokine network, with intertwined effects between disparate cytokines and cell types. Furthermore, some of the cytokines sharing the γc receptor (i.e. IL-2, IL4, IL-7, IL-9, IL-15 and IL-21) elicit surprisingly similar responses to the inflammatory cytokines (Lee et al. 2025[175]). A comparison of the effects of sepsis on organ gene expression to those of 6 singles and 15 pairs of recombinant cytokines was achieved in mice. Mechanistically, the authors map the cellular effects of sepsis and cytokines by computing changes in the abundance of 195 cell types across 9 organs, which they validate by whole-mouse spatial profiling. Strikingly, the authors found that the pairwise effects of tumor necrosis factor plus IL-18, or IFN-γ or IL-1β suffice to mirror the impact of sepsis across tissues. This work decoded the cytokine cacophony in sepsis into a pairwise cytokine message capturing the gene, cell and tissue responses of the host to sepsis (Takahama et al. 2024[304]).

### Compartmentalization 

#### Concomitant pro- and anti-inflammatory responses

In 2001, we postulated that “SIRS and CARS should not be opposed, but most probably occur concomitantly in different compartments: SIRS predominates within the inflamed tissues while blood leukocytes show hypo-reactivity”. This statement was accompanied by a figure illustrating that the pro-inflammatory and the anti-inflammatory response were concomitant (Cavaillon et al. 2001[52]). But the tenors in the field continued to state that the pro-inflammatory response was preceding the anti-inflammatory response (Hotchkiss et al. 2009[135]). It was only in 2013, twelve years after our claim, that a figure displaying a concomitant occurrence was finally proposed in a high rank journal (Hotchkiss et al. 2013[136]). Indeed, thinking outside the box may only lead to consensual agreement once the tenors in the field will have made the idea their own (Cavaillon 2023[47]). Nowadays, a consensus has been reached and the idea of two consecutive steps is not supported anymore, and the concomitant occurrence of both pro- and anti-inflammatory response prevails. 

#### A tissue specific response

In our 2001 statement, we also pointed out the concept of compartmentalization which was associated with the idea that the immune status of sepsis patients cannot be defined only based on the properties of the immune cells within the blood stream. Accordingly, a deeper understanding of compartmentalized immune responses and the dynamic immune landscape in sepsis is critical for developing precision therapies (Garduno et al. 2024[111]). A recent analysis of all immune cells within the whole body further illustrates that blood leukocytes are far from being the most representative immune cells. All together there are 1.8 x 10^12^ immune cells, the vast majority being in bone marrow and in lymphatic system. For example, there are more immune cells in the gut, the lung and twice as many immune cells in the skin than in the blood (Sender et al. 2023[268]).

The immune status of leukocytes in blood and in tissues has been rarely compared. The CD24 expression on circulating and airway neutrophils was found similar (Skirecki et al. 2016[285]). A high expression of HLA-DR on monocytes from the alveolar compartment was noticed as compared to the blood (Skirecki et al. 2016[285]; Bendib et al. 2021[22]). Since low HLA-DR expression is a hallmark of immunosuppression, these observations trend to favor the idea that there is no such altered immune status for the monocytes present within the lungs. Furthermore, in contrast to blood monocytes, alveolar macrophages cannot be rendered tolerant to LPS (Philippart et al. 2012[241]). This phenomenon is consecutive to a cross-talk between alveolar macrophages and alveolar epithelial cells. Upon LPS stimulation, alveolar macrophages secrete TNF that induces the release of GM-CSF by alveolar epithelial cells. GM-CSF prevents induction of endotoxin tolerance and potentiates the response of the alveolar macrophages (Jiang et al. 2025[152]). Thus, the tissue microenvironment determined phenotypic adaptions following remote injury and there is an organ specific inflammatory transcriptomic program change. During experimental sepsis, the number of differentially expressed genes in lung macrophages is extremely low compared to other organs, kidneys and liver harboring the highest expression of genes (Hoyer et al. 2019[139]). Myocardial infarction and stroke induce a lower transcriptomic activity in liver and kidney macrophages as compared to sepsis. Local proliferation or rather recruitment explain the expanding tissue macrophage numbers. Skirecki et al. (2022[282]) confirmed the compartmentalization of the monocyte response in a porcine model of endotoxin shock, highlighting the importance of studying tissue-resident cells in addition to their circulating counterparts. They showed that 100 % of monocytes present in broncho-alveolar lavage fluids (BALF) expressed the porcine MHC II antigen (swine leukocyte antigen, SLA-DR), and expression was higher compared to peripheral blood and bone marrow. The mitochondrial membrane potential (ΔΦ), an early indicator of apoptosis, displayed by CD14+CD163+ in BALF was lower than in peripheral blood and bone marrow monocytes. Measurements of cytokines in tissues strongly suggest their local production. For example, human lungs are a major source of IL-6 and IL-8 (Tyburski et al. 2001[315]; Bendib et al. 2021[22]; Brusletto et al. 2022[32]). High levels of the anti-inflammatory cytokine, IL-10, have been regularly measured in plasma from patients with sepsis, correlating with plasma levels of IL-6, IL-8 and TNF (Van Deuren et al. 1995[318]). In contrast, much lower quantities of IL-10 are detected in different tissues as compared to blood levels. This may imply that interactions between pro- and anti-inflammatory molecules operating at tissue levels are quantitatively different in organs compared to the circulation (Brusletto et al. 2022[32]). Another illustration of the discrepancy between organs and the blood compartment is the analysis of the CCL5 chemokine (RANTES). Its presence is decreased in the blood of septic patients, probably as a reflection of thrombocytopenia, since platelets are a main source of plasma RANTES (Cavaillon et al. 2003[53]). In contrast, in a pig model of endotoxemia, an increased transcription of RANTES was observed in lung and liver (Hellerud et al. 2025[129]). A further demonstration of this discrepancy was achieved in a murine CLP model: the *ex vivo* TNF production upon LPS stimulation is reduced in peripheral blood mononuclear cells and in splenic macrophages, but is enhanced in alveolar macrophages and Kupffer cells (Suzuki et al. 2006[302]). Other examples have been recently reviewed (Cavaillon et al. 2024[54])

Different studies have analyzed the transcriptomic profile in organs of patients who died of sepsis. Thousands of protein-coding and non-coding RNA transcripts were modulated in lungs, hearts, spleens, livers and kidneys (Brusletto et al. 2020[33]). The authors identified specific biomarker panels both protein-coding and non-coding RNA transcripts, which differed from organ to organ. Septic shock is an extremely heterogeneous disorder, not only when different individuals are investigated, not only in terms of pathogens but also when comparing different tissues of the same patient. Indeed, in a study investigating the gene expression in colon, heart, kidney, lung, cortex and hippocampus, it was observed that gene coding for different key pathways of immune response, oxidative damage, mitochondrial dysfunction, senescence, RNA and amino-acid metabolism could be turned on or off depending on the organ and even within the tissue compartments when considering the brain (Pinheiro da Silva et al. 2023[245]). For example, the gene coding for Complement factor B was turned off in lung but turned on in colon; the gene coding for IL-1 receptor (ST2) was turned off in lung but turned on in brain cortex and heart; the gene coding for CD14 was turned off in lung and kidney but turned on in brain cortex and colon. In a porcine model of LPS injection, resulting in death within 5-10 h, gene expression analyzed in the brain revealed significant differential expression correlating with endothelial cell disruption, immune/inflammatory alterations, and potential alterations in microglia. The main downregulated immune response genes include: OCL (Occludin), SLC19A3 (thiamine transporter), SLC52A3 (riboflavin transporter), SOX18 (transcription factor), and FOXF2 (transcriptional regulator), while main upregulated genes include: ATF3 (transcription factor), IRF1 (transcription factor), JUNB (Transcription factor), ICAM1 (adhesion molecule), CXCL10 (chemokine), and ADM (Adrenomedullin) (Neill, 2025[220]).

Sepsis is associated with specific alterations within the bone marrow. In humanized murine model, CLP sepsis and endotoxemia induced a similar expansion and proliferation of early hematopoietic stem and progenitor cells in the bone marrow, while committed progenitors decreased (Skirecki et al. 2015[284]). In a murine CLP model, analysis of activated caspase-1/-3/-7 revealed an increased apoptotic (by 30 %) but not pyroptotic signaling in the bone marrow hematopoietic stem cell of mice predicted to die. The bone marrow from these mice revealed spikes of IL-6, CXCL1/KC, CCL3/MIP-1a, and CCL2/MCP-1 as compared to mice predicted to survive and controls (Skirecki et al. 2021[283]). Skirecki et al. (2020[286]), demonstrated that the bone marrow is the primary site of CD4+ memory T cell homing and proliferation after sepsis-induced lymphopenia and identified IL-7 as the major inducer of proliferation of these memory CD4+ T cells. 

Furthermore, the bone marrow is the witness of a key feature of sepsis, namely hemophagocytic lympho-histocytosis, also known as macrophage activation syndrome. It occurs in 60-65 % of sepsis patients (François et al. 1997[105]; Stéphan et al. 1997[291]). Ferritin was proposed as a diagnostic and surrogate biomarker. Interleukin-1 receptor antagonist (IL-1ra, anakinra) appears as an effective treatment for sepsis and COVID-19 patients who display such a hemophagocytic lymphohistocytosis (Shakoory et al. 2016[273]; Dimopoulos et al; 2020[91]).

## Sepsis at the Individual Level

### Animal experimental models 

*“There is a lot of animal in humans, but not a lot of human in animals“*

Stefan G. Hofmann *(German clinical psychologist)*.

Sepsis gathers numerous different settings such as tremendous genetic diversity of the human population, differences of age and gender, source of infection, nature of the pathogens, rarely recapitulated in animals. As we previously pointed out, despite having saved thousands of animals over the last three decades in multiple preclinical studies, no new effective drug has emerged that has clearly improved patient outcomes. The reasons for this failure are the use of irrelevant animal models (Cavaillon et al. 2020[58]). One of the major drawbacks of sepsis translational research is the use of animal species, such as mice, rats and baboons, which are highly resistant to infection and endotoxin in contrast to humans. The blood transcriptomic response to an LPS challenge as well as plasma proteomes and lipidomes in six LPS-sensitive species (rabbit, pig, sheep, cow, chimpanzee, human) have been compared to LPS-resilient species (Gregory et al. 2024[122]). After LPS stimulation, among the gene responses that most differ between sensitive and resilient species are a relatively small number of genes that correspond to LPS detoxification, TLR response, bacterial control, and apoptosis. 

The experts in the field recommended that challenge with LPS should not be considered as an appropriate model for recapitulating human sepsis (Libbert et al. 2019[189]), and invited scientists to use antimicrobial agents, as it is a key management of human sepsis (Hellman et al. 2019[130]). Indeed, so far, antibiotics have been poorly used, with a frequency below 20 % of the CLP papers published between 1999 and 2019 (Joffre, 2023[153]). Furthermore, an interesting discrepancy in the murine model of CLP has been reported with the influence of seasons on male survival (Garcia et al. 2023[110]). 

The use of sensitive animals such as the rabbit should be encouraged as it has been shown to help to predict clinical efficacy of new antibacterial drugs for the treatment *of P. aeruginosa* pneumonia (Gras et al. 2023[121]). Among other sensitive animals, pigs have various recognized advantages, reproducing important immunological features with patients. The inflammatory responses measured at the transcriptomic level in lungs, kidneys, liver and spleen in patients with fulminant meningococcal sepsis were reproduced in the porcine model of the disease, although some differences existed regarding the top-upregulated factors in individual organs (Brusletto et al. 2022[32]). Rhesus macaques have been used to mimic human sepsis as recently reported for *Klebsiella pneumoniae *induced shock (Strich et al. 2025[294]). But these non-human primates are highly resistant to infection and high doses of bacteria were needed (from 2.4 x 10^7^ to 3.3 x 10^9^ CFU/kg). 

### Patient heterogeneity and Endotypes 

Sepsis is a systemic disorder associated with organ specific features. Accordingly, the immune status of the patients cannot be oversimplified by a simple analysis of blood leukocytes, and a simplistic approach to boost the immune system might not be appropriate for all patients (Cavaillon et al. 2014[56]). It has been proposed that multiple immune and non-immune-related parameters, including clinical and microbiological data, be integrated into diagnostic with the aid of machine learning and artificial intelligence technique, aimed to defined different endotypes. Specific immunological biomarkers and high-throughput omics-based techniques provide novel insights into immune dysregulation and allow the identification of sepsis endotypes, a prerequisite to apply precision medicine and improve patient outcomes (Cajander et al 2024[35]; Giamarellos-Bourboulis et al. 2024[114]). Furthermore, deciphering the different endotypes can help to identify target drugs for a stratified treatment of sepsis (Sun et al. 2023[298]; Kalil et al. 2025[155]). 

Four different phenotypes (named α, β, γ, δ) associated with different outcomes were initially described based on 29 variables including demographic variables, vital signs, markers of inflammation, markers of organ dysfunction or injury and serum levels of glucose, sodium, hemoglobin, chloride, bicarbonate, lactate, and albumin (Seymour et al. 2019[271]). Implementing only six-parameters (creatinine, lactate, aspartate transaminase, bilirubin, C-reactive protein, and international normalized ratio), the presence of the four phenotypes across cohorts of bacterial sepsis could be validated and extended to COVID-19 patients (Karakike et al. 2024[157]).

Stratification using monocytic HLA-DR levels, frequency of immature neutrophils rates and levels of plasma IL-10 have allowed to define a high-risk group of patients with an increased rate of ICU-acquired infections, significantly higher mortality and longer ICU length of stay (Bodinier et al. 2023[23]). Gene expression signatures have also been used to predict severity, organ dysfunction and mortality in both emergency department and ICU patients. In a study involving patients from the Netherlands and UK, genome-wide blood gene expression profiles from admission samples, led to identify four molecular endotypes for sepsis, designated Molecular Diagnosis and Risk Stratification of Sepsis (MARS) 1 to 4. A 140-gene expression signature reliably stratified patients with sepsis to the four endotypes. Only Mars1 was consistently significantly associated with 28-day mortality across the cohorts (Scicluna et al. 2017[265]). *Bisphosphoglycerate mutase* (BPGM): *transporter 2, *and* ATP binding cassette subfamily B member* (TAP2) reliably identified patients with a Mars1 endotype. In another investigation, patients with early sepsis could be stratified into five distinct endotypes, named Neutrophilic-Suppressive (NPS), Inflammatory (INF), Innate-Host-Defense (IHD), Interferon (IFN), and Adaptive (ADA). Significant differences were observed in terms of outcome. There were two severe groups (NPS: mortality = 43 %; and INF: mortality = 25 %) and one relatively benign (ADA survival = 100 %) (Baghela et al. 2022[12]). Others have identified three distinct endotypes with different survival rates (Tang et al. 2024[311]). Similarly, three endotypes termed “Inflammopathic”, “Adaptive”, and “Coagulopathic” have been validated in 9 independent datasets from 5 different countries (n=600). The Adaptive subtype is associated with a lower clinical severity and lower mortality rate, and the Coagulopathic subtype is associated with higher mortality and clinical coagulopathy (Sweeney et al. 2018[303]). A molecular phenotyping applied in multiple sepsis cohorts, allowed to classify patients as hypo-inflammatory and hyperinflammatory. Interestingly, the hyperinflammatory phenotype better responded to activated protein C, while in the other group, activated protein C had a pejorative effect (Sinha et al. 2023[281]). A differential gene analysis was carried out in West Africa, Southeast Asia, and North America. The authors identified four sepsis subtypes: immunocompetent, immunosuppressed, immune-metabolic and acute inflammation. The low mortality immunocompetent group is specified by features that describe the adaptive immune system. In contrast, the three high mortality groups show elevated clinical severity consistent with multiple organ dysfunction (Chenoweth et al. 2024[68]). In children with sepsis, the transcriptomic analysis revealed major differences between age groups defined as neonates (≤28 d), infants (1 month to 1 year), toddlers (2-5 years) and school age (≥6 years) and age-matched controls. In contrast to the largely upregulated transcriptome in all other groups, neonates exhibited a predominantly downregulated transcriptome when compared with controls (Wynn et al. 2011[341]). Because gender and sexual hormones have an impact on the pathophysiology of sepsis and its outcome (Lakbar et al. 2023[171]), we suggest that these transcriptomic analyses and the associated endotypes would be revised considering the individual gender of each patient.

### Microbiome and sepsis 

Nowadays, it is impossible to conceive any biological functions from the toes to the brain without mentioning the role played by the gut. It has been a long time since scientists considered the influence of the gut on human beings. Elie Metchnikoff was among the first to study gut microbiota. He defined the bacteria of putrefaction within the gut, and identified their capacity to produce poisons (Metchnikoff, 1908[210]). Among them, he identified different metabolites produced by these bacteria capable of inducing tissue damages similar to those found in elderly humans (Metchnikoff, 1910[209]). He favored the concept of autointoxication as did before him Charles Bouchard, and after him Charles Alfred, Sir William Arbuthnot Lane and John Harvey Kellogg. Years later, the gut was again pointed out as the culprit and considered as the motor of multiple organ dysfunction syndrome (MODS) (Carrico et al. 1986[44]). The gut dysfunction is consecutive to an alteration of its epithelial barrier which favor bacterial translocation, and a disbalance between protective bacteria and pathogenic ones (Barlow et al. 2022[17]). Accordingly, selective digestive decontamination has been proposed for more than 40 years, offering pro and con debate, while no consensus has still emerged (Carlet, 2024[39]).

The microbiota dysbiosis is characterized by an alteration of symbiotic flora and elevated abundance of Enterococcus. Additionally, patients with a high burden of Bacteroides, especially *B. vulgatus*, had a higher acute physiology and chronic health evaluation II (APACHE II) score and longer stays in the intensive care unit (Sun et al. 2023[299]). An association between certain gut bacteria and the presence of sepsis has been reported, while an association of other bacteria with survival has been observed (Yang et al. 2024[349]). Microbial compounds, such as short-chain fatty acid, perform a crucial task in modulating the inflammatory response and maintaining intestinal barrier function. However, the role of other microbiota components, such as viruses and fungi, in sepsis will need further investigations (Piccioni et al. 2024[242]).

In an experimental approach of sepsis following injection of *Serratia marcescens *and* Klebsiella oxytoca *in mice, it has been reported that the gut microbiota improves survival in sepsis via the production of tryptophan-derived metabolites that activate the aryl hydrocarbon receptor (AhR) in macrophages inducing a transcriptional reprogramming and increasing bacterial clearance. Oral or systemic tryptophan supplementation increased survival. However, this protective mechanism is inhibited by enterobactin, a pathogen derived siderophore, revealing a microbial interplay (Keskey et al. 2025[162]). In another murine model in which mice underwent a 30 % liver excision and received human pathogens (Tetracycline resistant *Enterococcus faecalis*, multidrug resistant *Klebsiella oxytoca*, multidrug resistant *Serratia marcescens *and *Candida albicans*), a fecal microbiota transplant prepared by harvesting the cecal contents of normal healthy mice protected the mice. While pathogen infection drives butyrate deficiency, the protective effect of the fecal transplant was linked to the expansion of butyrate-producing *Bacteroidetes* (Kim et al. 2020[164]). Of note, a dysbalance has been also observed in the lung microbiota of patients with pneumonia, with an enrichment of *Enterobacteriaceae *and other *Proteobacteria *(Kritikos et al. 2025[169]). Indeed, it has been shown that microbiota and immune dynamics during acute critical illness were highly interconnected and dominated by intestinal *Enterobacteriaceae* enrichment which was coupled with impaired innate antimicrobial effector responses, including hypofunctional and immature neutrophils and was associated with an increased risk of nosocomial infections by various bacterial and fungal pathogens (Schlechte et al. 2023[277]).

Most interestingly, it has been demonstrated these recent years that in addition to infection, stress can be a major player modulating gut microbiome (Zhang et al. 2024[362]). One can hypothesize that the stress undergone by the patients in ICU can thus also contribute to the microbiota dysbiosis. It has been reported that secretions of mucin from the duodenal glands of Brunner enhance host defense by promoting gastrointestinal *Lactobacilli *proliferation. Central amygdala neurons stimulate Brunner's glands via vagal nerves. Chronic stress inhibited a brain-vagus nerve circuit that stimulates glandular secretion, thereby suppressing *Lactobacillus* populations and systemic immunity (Chang et al. 2024[59]). A cross-talk between gut - immune system and brain has been demonstrated in a restraint stress murine model. The stress induced a gut leakage and microbiota exposure, leading to an intestinal Th17 cells expansion, which contributes to elevated systemic IL-22. IL-22 gained access to the septal area of the brain and directly suppressed septal neuron activation, preventing stress-elicited anxiety (Xia et al. 2025[342]). 

Dysbiosis induced by trauma and sepsis persists up to 14 to 21 days after onset and is sex-specific. Further subgroup analyses based on sex revealed resistance to changes in microbiome diversity among female trauma patients compared to healthy males. Sex-specific changes in fecal metabolites were also observed after trauma and sepsis, while plasma metabolite changes were similar in both males and females (Munley et al. 2024). Addressing the patients' microbiome is a recent concept, and further investigations are expected to fully appreciate whether its manipulation may lead to better outcome.

## Old and New Cares for Sepsis

### Antibiotic therapy

Antibiotics are clearly the most important part of the therapy of sepsis, which is a severe infectious disease (Dellinger et al. 2013[88]). They should be started as soon as possible, without any delay. One up to 3 hours are often proposed in the guidelines. Indeed, the rapid completion of a 3-hour bundle of sepsis care and rapid administration of antibiotics, but not rapid completion of an initial bolus of intravenous fluids, were associated with lower risk-adjusted in-hospital mortality (Seymour et al. 2017[270]). A very important point is that samples (blood, urines, bronchi, peritoneum, CRL…) must imperatively be performed before starting antibiotics. If not, the bacteriology is likely to be negative. Then, the initial empiric antibiotic therapy must be blindly continued. The quality of this therapy is far lower, the length of therapy will be very difficult to decide, and any de-escalation of the initial therapy becomes impossible. In too many cases, such a strategy is not part of the educational package of students, and therefore is not applied in practice. This needs an excellent organization, and a good laboratory, sometimes difficult to set up in small hospitals.

#### Antibiotic strategies during sepsis

Many recommendations have been published regarding this issue (Rhodes et al. 2017[256]; Evans et al. 2021[98]). Most of them propose to use antibiotics (or combinations) with a very large spectrum of action, and covering resistant, or poly-resistant bacteria, in particular using very recent compounds. The risk of this kind of behavior is to increase AMR. In addition, it is often not necessary, nor appropriate. An important proportion of sepsis cases are community acquired, and therefore not due to very resistant bacteria. While early, appropriate antibiotic therapy is important, the duration of treatment remained uncertain. In a recent study involving hospitalized patients with bloodstream infection (55 % of patients in the ICU and 45 % on hospital wards) and with infections originating from the urinary tract (42.2 %), abdomen (18.8 %), lung (13.0 %), vascular catheters (6.3 %), and skin or soft tissue (5.2 %), antibiotic treatment for 7 days was not inferior to treatment for 14 days (Daneman et al 2025[81]).

The level of bacterial resistance is very different from a microorganism to another, and from country to country. Therefore, proposing universal guidelines is not realistic. Let's have a few examples.

#### Antibiotic strategies during sepsis-associated pathologies

A severe community-acquired pneumonia can be due of course to *Pneumococcus*, more rarely to *Staphylococcus*, *Streptococcus*, *Mycoplasma pneumoniae*, or *Legionella pneumophila* (during specific situations). In those cases, provided the patient has not been hospitalized recently, or is coming from long term care facilities, and is not immuno-depressed, the use of broad-spectrum antibiotics is not needed at all, and could be sub-optimal. Looking at pneumococcal markers can help a lot. Penicillin is far more active against pneumococcus than very recent compounds, which are too often used. If, in this area, staphylococci are highly resistant to methicillin, then another antibiotic therapy is needed, in particular vancomycin.

A sepsis due to peritonitis can be treated easily with the combination of piperacillin / tazobactam, switching to amoxicillin / clavulanic acid if all bacteria are susceptible to this drug. There is no need to add metronidazole, since those two drugs have an excellent activity against anaerobes. 

If sepsis is from nosocomial origin, then, a broad-spectrum antibiotic therapy is needed. However, the choice of the compounds depends on the level of AMR in the given country. In all cases, bacterial samples must be performed as soon as possible, before starting antibiotics. This will allow to de-escalate antibiotic therapy very quickly. This is a very important point. A combination therapy is often prescribed. In most cases, the second drug, often an aminoglycosides, can be stopped after identification of the various involved micro-organisms. The fact that sepsis is nosocomial does not authorize extravagant therapies.

In conclusion, each case needs some reflections from the health care professionals, before prescribing, since there is no universal and automatic antibiotic therapy to treat sepsis.

### Therapies supposed to act on some of the many mechanisms involved in sepsis

Since the initial paper published 33 years ago, offering the first consensual definition of “sepsis syndrome” (Bone et al. 1992[24]), a huge number of papers, controversies and pro-con debates have been published, and discussed during many international meetings. Most of those excellent studies, either randomized controlled trials, or meta-analyses, failed to show any significant reduction in mortality of this syndrome, and of ARDS, when using only one drug (Cavaillon et al. 2020[58]). 

#### Corticosteroids for sepsis

The use of steroids during sepsis, septic shock and ARDS is one of the most important, and ongoing controversy in intensive care medicine, lasting since 40 years. This induced many conflicts, and pro/con debates. Some well-known scientists were convinced that steroids work during sepsis and ARDS (Meduri et al. 1998[208], Sprung et al. 1999[289] & 2008[288]). This has been the case for corticosteroids, either when using initially very high doses, or later using low doses. However, a significant efficacity was shown by Annane et al. (2018[8]) during septic shock, when mineralosteroids were added to corticosteroids. Therefore, only this indication was retained in the “sepsis surviving guidelines”. The destiny of steroids to treat severe infections totally changed when the pandemic with COVID-19 happened, in 2000. Many cases of this viral infection were extremely severe, and deadly. The RECOVERY study was performed very urgently in Great-Britain, looking at the efficacy of many different drugs (Horby et al. 2021[133]). In this study, corticosteroids were efficacious, in particular in the most severe patients. A meta-analysis, promoted by WHO, was also in favor of steroids (Shankar Hari et al. 2021[274]). However, those studies had very serious methodological weaknesses, and a confirmation is mandatory, if severe cases of COVID-19 happen again (Carlet et al. 2020[41] & 2025[42]). It is important to note that methylprednisolone was the only steroid inducing positive results, when used alone. It is likely that steroids are nowadays widely used, even in non-COVID cases of bacterial sepsis. It is a serious issue, since the ratio efficacy/safety of the various compounds has been poorly evaluated. A multi-centric study, looking specifically at this point is urgently needed. Finally, a worldwide epidemiological study, looking at the usage of corticosteroids for severe infections, and to a possible overuse of those drugs, would also be important to perform. 

The exact nature of the employed steroids might be of great importance. For example, in a cohort study including 88,275 adult patients with septic shock, for those who began hydrocortisone treatment, the addition of fludrocortisone was superior to hydrocortisone alone (Bosch et al. 2023[25]; Annane et al. 2018[8]). Improvement of corticoid delivery could be proposed such as the use of micelles to encapsulated dexamethasone (Louaguenouni et al. 2025[193]). Like most of the clinical investigations conducted so far, the use of corticoids could be greatly improved by precision medicine. Defining endotypes associated with adults with sepsis responsiveness to corticosteroids could help to a better inclusion of patients who could respond to the treatment (Fleuriet et al. 2023[103]). Individual-specific glucocorticoid receptor binding could be traced to single-nucleotide polymorphisms (SNPs) that altered the binding motifs of glucocorticoid receptor or its cooperating factors, while another set of genetic variants modulates dexamethasone response through affecting chromatin accessibility or chromatin architecture. Several SNPs affect dexamethasone-regulated glucocorticoid receptor binding and gene expression-controlled dexamethasone-driven metabolic perturbations. Accordingly, knowledge of the genetic variants that predispose individuals to metabolic side effects allows for a precision medicine approach for the use of clinically relevant glucocorticoids (Hu et al. 2021[141]).

#### Steroids for ARDS

Although many studies performed in the last decades were negative, some recent data show that corticosteroids are probably efficient during ARDS. The guidelines have already, and may-be too early, adopted this indication. However, additional data will be more than welcome (Leung et al. 2025[177]).

#### Steroids for severe community acquired pneumonia 

Similarly to ARDS, some convincing data have been published recently, and their usage is already proposed in recent guidelines (Leung et al. 2025[177]). We hope that it will be confirmed by further studies. 

#### Combination of steroids and anti-IL-6 RI 

Although appropriate studies have not yet been performed, it is important to mention that some data coming from the studies performed during severe cases of COVID-19 (Shankar-Hari et al. 2021[274]) showed that the effect of IL-6-RI alone was very limited. Only patients treated with the two drugs were positive. Therefore, this combination might be efficient in severe cases of COVID-19. This needs to be confirmed.

#### Old and new targets

Translational research is fueling the field of sepsis with new drugs aimed to target new pathways or revisiting old drugs, with putative new hopes. Sometimes, sophisticated studies are proposed to decipher the mechanistic aspect of the drug. However, in many reports the percent of saved animals remains pretty low. For example, targeting mitochondrial copper with supformin (LCC-12), a dimer of metformin has been proposed. It induces reduction of the NAD(H) pool, leading to metabolic and epigenetic states that oppose macrophage activation. While it saved 100 % of the mice challenged with LPS, it had a very modest effect in mice exposed to 100 % lethal CLP, ending to 20 % survival (Solier et al. 2023[287]). As we pointed out earlier, LPS injection should not be employed to mimic sepsis. It may raise false hopes as apparent successes have been obtained with such models with apoptotic body based biomimetic hybrid nanovesicles (Lan et al. 2024[172]), or reverse transcriptase inhibitors (Hullahall et al. 2025[143]). Similar limited effects in the CLP murine model was obtained with an anti-miR-93-5p therapy (Dragomir et al. 2023[94]), a reconstituted high-density lipoprotein therapy (Tanaka et al. 2020[307]), an exosome-based super-repressor IκBα (Exo-srIκB) delivery, a new concept of NF-κB inhibitor (Park et al. 2024[231]), or with a tripeptide, Arg-Lys-His, derived from *Akkermansia muciniphila*, a commensal gut bacterium that reduces inflammation, and which may act as a novel endogenous antagonist for TLR4 (Xie et al. 2023[344]). Nevertheless, despite similar limited effect in a rat model of sepsis (Cheng et al. 2024[67]), XueBiJing, a Chinese herb-based intravenous formulation has moved to a randomized clinical trial and displayed a certain efficiency to reduce sepsis mortality of patients from 26.1 % to 18.8 % (Liu et al. 2023[191]). 

A preclinical investigation targeting TREM-1 moved mortality in a CLP murine model from 80 % to 50 % (Bouchon et al 2001[26]). A recent clinical trial has investigated nangibotide, a TREM-1 inhibitor. This trial did not achieve the primary outcome of improvement in SOFA score at the predefined sTREM-1 value. In the overall population (n=354) the mortality was in the placebo, the low dose of nangibotide and high dose, 25 %, 32 % and 25 %, respectively. In patients with high levels of plasma soluble TREM-1 the mortality was 31 %, 39 % and 28 %, respectively, but this study was not powered to detect mortality differences. Whether nangibotide at higher concentrations of TREM-1 activation could be beneficial will need further studies (François et al. 2023[104]).

In human patients, aspirin could reduce the mortality risk of sepsis-associated encephalopathy patients. However, patients receiving high-dose of aspirin exhibited a higher mortality risk compared to those in the low-dose group (Huang et al. 2025[142]). These results are reminiscent of a previous study which established that in sepsis patients, acetaminophen use was associated with mortality (Lee et al. 2012[173]). Indeed, fever illustrates the yin yang aspect of the host response, because in sepsis appropriate fever (37.5°C - 38.4°C) was protective while excessive fever (≥39.5°C) and no fever were deleterious. Most interestingly, therapeutic hyperthermia artificially raising body temperature through external warming, was associated with improved survival in afebrile critically ill patients with sepsis (Drewry et al. 2022[95]).

Many clinical studies targeting inflammatory cytokines, especially IL-1 and TNF, have been performed, in the past, with negative results (Cohen & Carlet, 1996[71]) despite very promising results obtained in preclinical sepsis models. More recently, particularly to address severe COVID-19, IL-6 and its receptors have been proposed and tested as targets. The rational of these approaches were rarely offered, knowing that IL-6 possesses both pro- and anti-inflammatory properties (McElvaney et al. 2021[206]). Indeed, as we pointed out in 2001, IL-6 is the most ambiguous cytokines (Cavaillon, 2001[50]), and injection of IL-6 has never killed any mice! In fact, injections of huge amounts of IL-6 (10 µg) in mice protected them against a lethal challenge of LPS. Thus, the question whether the high plasma levels of IL-6 observed in sepsis patients are just a marker of severity and eventually of outcome, or a player of the detrimental response, remains to be addressed in bacterial sepsis. Nevertheless, some pre-clinical studies have concluded on the protective effects of IL-6 neutralization. Selective inhibition of IL-6 trans-signaling by soluble gp130-Fc rather than complete blockade of IL-6 signaling by anti-IL-6 significantly improved survival after CLP sepsis and completely prevented intestinal epithelial cell apoptosis (Barkhausen et al. 2011[16]). In accordance, antibodies to interleukin-6 were able to ameliorate gastro-intestinal motility, suppress inflammation and normalize the permeability of the colonic wall, with the preventive administration (Nullens et al. 2016[224]). In addition, soluble gp130 reduced sepsis associated inflammation, preserved blood brain barrier integrity, modulated monocytes/macrophages cells and lymphocytes transmigration and activation, and ensuing cognitive decline (Jiang et al. 2023[151]). When combined with anti-PD-1, IL-6 blockade reduces neutrophil infiltration, lymphocyte apoptosis, and bacterial burden while preserving tissue integrity in a CLP model of sepsis. However, the improvement in survival was not statistically significant (Lee et al. 2025[176]).

IL-6 has thus been targeted in severe COVID-19 cases. As revealed by a meta-analysis, anti-IL-6 receptor gp80, Sarilumab and Tocilizumab, did not reduce the risk of requiring mechanical ventilation, but were slightly although significantly beneficial in reducing the risk of clinical worsening and the risk of mortality (Jafari Abarghan et al. 2024[150]). Another meta-analysis concluded that the administration of IL-6 antagonists, compared with usual care, was associated with lower 28-day all-cause mortality. However, a figure provided as a supplement (!) showed that only the open-label studies were positive altogether, while quite a large number of individual studies were negative, and no effect was reported in the placebo-controlled trial (Shankar-Hari et al. 2021[274]). An aspect which has not been addressed is why to use antibodies targeting the gp80 receptor of IL-6 rather the cytokine itself. Of note, gp80 receptor (IL-6RI) exists as a membrane form and as a soluble form. Because the soluble gp80 allows cells missing this receptor, but harboring the gp130, to respond to IL-6, one can hypothesized that these given antibodies prevent specific cells that remain to be identified, from being activated and to play a deleterious role. Because, most COVID-19 patients, in the many studies performed with anti-IL-6 RI were also treated with corticosteroids, a careful analysis is highly desired (Carlet, submitted[38]).

Among the plethora of activities of IL-6, let's mention its capacity to favor coagulation via its induction of thrombin as illustrated by the capacity of anti-IL-6 antibodies to attenuate coagulation activation in experimental endotoxemia in chimpanzees (van der Pool et al. 1995[317]). This is another example of the yin yang response during sepsis. Coagulopathy is a hallmark of sepsis and disseminated intravascular coagulation is a frequent complication. It is characterized by activation of coagulation, excessive intravascular fibrin formation, and vascular thrombosis resulting in organ hypoperfusion that may contribute to organ failure (Nimah & Brilli, 2003[222]). But coagulation is also a mean to fight infectious agents, and to prevent their dissemination (Echtenacher et al. 2001[97]). For example, Factor XII-driven coagulation enhances innate immunity by trapping pathogens and restricting bacterial infection in mice as illustrated in *Streptococcus *pneumoniae and *Staphylococcus aureus* skin infection (Nickel et al. 2025[221]). This ambivalent property may explain why targeting coagulation in human sepsis with anti-thrombin antibodies, recombinant tissue factor inhibitor, and eventually activated protein C have failed.

As mentioned above, most bacteria express or secrete toxins, sometimes very aggressive, like endotoxin of entero-bacteriacaes, or exotoxins of *streptococcus *and *staphylococci*. Studies performed a long time ago with anti-endotoxin antibodies were initially positive by the authors, but only in sub-groups (Ziegler et al. 1991[370]), and those initial results failed to be confirmed, because the employed antibody failed to neutralize the endotoxin (Warren et al. 1993[332]). A mean to circumvent these difficulties would be the use of antibodies or vaccines targeting either O polysaccharides or the LPS core regions (Cross 2023[76]).

### Immunomodulation

As mentioned earlier, altered immune status is a hallmark of sepsis, when assessing the immunocompetence of circulating leukocytes. While an immunosuppression has been well recognized, the alteration of the immune cells might be quite different within the tissues, as illustrated by the macrophage activation syndrome observed within the bone marrow. This dichotomy should be precisely analyzed to offer personalized medicine, and thus appropriate immunostimulant treatment (e.g. IFNγ, GM-CSF, IL-7, anti-PD1 or PDL-1, thymosin α1) or immunosuppressive therapy (e.g. IL-1Ra). As we pointed out ten years ago, the immune status of leukocytes is not universally decreased, and their activated status in tissues contributes to organ failure. Accordingly, we were worrying that any immune-stimulatory therapeutic intervention could lead to potentially deleterious effects in some patients (Cavaillon et al. 2014[56]). Indeed, among mechanically ventilated patients with acute organ failure, treatment with interferon gamma-1b compared with placebo did not significantly reduce the incidence of hospital-acquired pneumonia or death on day 28. However, the trial had to be discontinued early due to safety concerns about interferon gamma-1b treatment (Roquilly et al. 2023[257]). Intravenous administration of recombinant IL-7 resulted in a two-threefold increase in absolute lymphocyte counts compared to placebo. The treatment reversed severe lymphopenia and was associated with increase in organ support free days. However, the study has been halted early because of three patients out of the fifteen who received the treatment, developed fever and respiratory distress. Inhaled bronchodilators, or non-invasive ventilation or intubation and mechanical ventilation allowed the problems to be solved rapidly (Daix et al. 2023[80]). In contrast, a similar approach in COVID-19 patients allowed an absolute lymphocyte counts increase but without a significant difference between IL-7 treated patients and placebo. Nevertheless, IL-7-treated patients had 44 % fewer hospital-acquired infections *versus* placebo-treated patients and no adverse effects were reported (Shankar-Hari et al. 2025[274]). Among immunostimulants, thymosin α1, is a 28-amino acid peptide produced in the thymus recognized for enhancing, and restoring immune function. Thymosin α1 acting on both myeloid and dendritic antigen-presenting cells, stimulates the adaptive immune response. A multicentric, double blinded, randomized, placebo controlled, phase 3 trial did not allow to establish clear evidence to suggest that thymosin α1 decreases 28-day all-cause mortality in adults with sepsis (Wu et al. 2025[338]). Using ferritin >4,420 ng/mL and <5,000 HLA-DR receptors/monocytes as biomarkers, patients were classified into macrophage activation-like syndrome (MALS; 20.0 %), immunoparalysis (42.9 %), and intermediate (37.1 %) with a respective mortality of 26.9 %, 47.6 % and 25.5 %. Unfortunately, the personalized therapy (IFNγ or IL-1Ra) did not allow to achieve different results than in the placebo group (Leventogiannis et al. 2022[178]).

### Blood purification

Extracorporeal blood purification therapies have been proposed as a possible adjunctive therapy to rebalance the immune response by removal of inflammatory mediators, such as cytokines, and/or PAMPs such as endotoxins (Waalders et al. 2025[323]). It consists of either filtration, diffusion, adsorption, or a combination of those. Polymyxin B-immobilized fiber column aimed to capture endotoxin (Toramyxin cartridge) is one of the most studied. A recent meta-analysis suggests that plasma exchange and polymyxin-B hemoperfusion may provide potential benefits for adult patients with severe infection or sepsis/septic shock when compared with standard care alone, but most comparisons were based on low or very low certainty evidence (Chen et al. 2023[61]). Few devices had some effects on ICU length of stay (e.g. haemoperfusion *cartridge* HA330), although none but Polymyxin B and plasma exchange had significant effects on mortality. Cares should be taken regarding the nature of the Gram-negative bacteria responsible of sepsis, as polymyxin B differs greatly in its capacity to interact with endotoxins depending on their bacterial origin (Cavaillon 2011[49]).

### Neuromodulation

A regulatory loop triggered by inflammatory cytokines, involving afferent vagal nerve and efferent vagal nerve, and the release of acetycholine and noradrenalin contribute to inhibit the production of inflammatory cytokines. The mechanism was termed the “cholinergic anti-inflammatory pathway” (Czura, 2025[78]). In clinical studies, triggering the vagal nerve with a specific implanted device allowed to improve patients with Crohn disease and rheumatoid arthritis. In a porcine model of sepsis, vagus nerve stimulation resulted in significantly attenuated multiple organ dysfunction and reduced vasopressor and fluid resuscitation requirements (Kohoutova et al. 2019[168]). In a mouse model of septic encephalopathy obtained after LPS injection, vagal nerve stimulation was achieved with noninvasive, high-frequency ultrasound. Mice subjected to the treatment demonstrated a modest, yet significant, improvement in survival rate, recovery from hypothermia, and increased locomotor activity. In septic mice, vagal nerve stimulation led to the restoration of aberrant firing patterns in hippocampal neurons. Additionally, proteomic analysis of hippocampal tissue in septic mice revealed abnormal increases in two proteins, tissue factor and acyl-CoA dehydrogenase family member 9 (ACAD9), which returned to control levels following the treatment (Imamura et al. 2023[148]). In human sepsis patients, vagal nerve activation can be achieved with acupuncture (Zhang et al. 2024[361]) or via transcutaneous auricular vagus nerve stimulation (Wu et al. 2023[340]). In the latter case, sepsis patients receiving the treatment experienced significantly lower serum pro-inflammatory cytokines and higher serum anti-inflammatory cytokines in sepsis patients as compared to sham stimulation. No differences in the APACHE II score and SOFA score were observed between the two groups. Altogether these studies suggest that vagal nerve stimulation could be an easy adjunctive treatment. 

## Conclusions

Despite tremendous and successful efforts to further decipher the mechanisms underlying sepsis, no new therapies have emerged during these last decades from preclinical investigation. Despite pertinent animal models, most studies have continued to use resilient animals to sepsis. Despite sepsis is a well-recognized systemic dysregulation, although the term could be inappropriate, no therapies have been specifically defined to target the organs specificities. In most clinical trials, inclusions entered different sources of infections, different types of pathogens, different levels of inflammation, different degrees of dysregulation, and a heterogenous population in terms of age, gender, underlying diseases, and genetic background. More targeted studies should be undertaken and the definition of endotypes should help to achieve “precision medicine” with the hope to identify sub-groups of patients for whom a given therapy could be beneficial. The role of bacterial toxins must be better investigated, since compounds with a significant activity against some toxins, used on top of antibiotics, could help to further decrease mortality of sepsis, and septic shock. It would also be necessary to think “outside the box”, in order to breake some certitudes, and define some new ways of research in the field of very severe infections, and sepsis.

## Declaration

### Conflict of interest:

The authors declare that they have no conflict of interest.

### Artificial intelligence:

The authors certify that they did not use AI for the writing of their review.

### Authors contribution:

Both authors contributed to the bibliography analysis, and the writing of the manuscript. 

### Dedication 

Jmc dedicates this review to Thomasz Skirecki (MD, PhD, Varsaw), his spiritual son who did so much to demonstrate the concept of compartmentalization. He died at the age of 37, in January 2025 (Osuchowski & Cavaillon, 2025[226]).
